# Postoperative pain outcomes following uniportal vs. multiportal video-assisted thoracoscopic surgery: a systematic review and meta-analysis

**DOI:** 10.3389/fsurg.2025.1689456

**Published:** 2025-11-10

**Authors:** Fahim Kanani, Alaa Zahalka, Moshe Kamar, Rijini Nugzar, Ory Wiesel, Yael Refaely, Anas Salhab, Mordechai Shimonov, Diego Gonzalez-Rivas, Firas Abu Akar

**Affiliations:** 1 Department of Surgery, The Edith Wolfson Medical Center, Holon, Israel; 2 Gray Faculty of Medicine, Tel Aviv University, Tel Aviv, Israel; 3 Department of Anaesthesia, The Edith Wolfson Medical Center, Holon, Israel; 4 Division of Thoracic and Esophageal Surgery, The Cardiovascular Center, Tzafon Medical Center, Affiliated with the Azrieli Faculty of Medicine, Bar-Ilan University, Ramat Gan, Israel; 5 Department of Cardiothoracic Surgery, Soroka Medical Center, Faculty of Health Sciences, Ben-Gurion University of the Negev, Beer Sheva, Israel; 6 Department of Thoracic Surgery, The Edith Wolfson Medical Center, Holon, Israel; 7 Department of Thoracic Surgery, Shanghai Pulmonary Hospital, Tongji University, Shanghai, China; 8 Department of Thoracic Surgery, Coruña University Hospital, Coruña, Spain

**Keywords:** uniportal VATS, multiportal VATS, postoperative pain, lung resection, meta-analysis

## Abstract

**Background:**

Video-assisted thoracoscopic surgery (VATS) has evolved from multiportal to uniportal approaches, theoretically offering reduced postoperative pain through single intercostal space access. However, inconsistent surgical definitions and mixed evidence have limited clinical guidance.

**Objectives:**

To systematically evaluate postoperative pain outcomes between true uniportal VATS (strictly defined by the 2019 European Society of Thoracic Surgeons criteria) and multiportal VATS for lung resections.

**Methods:**

We searched five databases during the period between January 2000 and January 2025 for comparative studies of uniportal vs. multiportal VATS reporting pain outcomes. True uniportal VATS requires a single intercostal incision (2.5–5 cm) with all instruments through one port. Meta-analyses were excluded to prevent data duplication. The primary outcome was 24-h pain intensity. A random-effects meta-analysis was performed to calculate standardized mean differences (SMD) with 95% confidence intervals (CI). Risk of bias was assessed using the revised Cochrane risk-of-bias tool (ROB 2) randomized controlled trials (RCTs) and the risk-of-bias in non-randomized studies of interventions (ROBINS-I) for observational studies.

**Results:**

Nineteen studies (6 RCTs, 13 observational) comprising 2,544 patients (1,156 uniportal, 1,388 multiportal) met the inclusion criteria. Uniportal VATS significantly reduced pain at 24 h (SMD −0.98, 95% CI −1.12 to −0.84, *p* < 0.0001), equating to a reduction of 2.5 points on a 10-point scale. Benefits persisted at 48 h (SMD −0.80) and 7 days (SMD −0.58). Opioid consumption decreased by 10.6 mg of morphine equivalents (95% CI −14.8 to −6.4). Heterogeneity was moderate (I^2^ = 63.6%). Studies using standardized analgesia protocols showed larger effects (SMD −1.05) with lower heterogeneity (I^2^ = 58.4%). Meta-regression identified a decrease in effect sizes over time (β = 0.05 per year, *p* = 0.024). Sensitivity analyses confirmed the robustness of the results, with all iterations maintaining statistical significance.

**Conclusions:**

True uniportal VATS provides clinically meaningful reductions in postoperative pain compared with multiportal approaches when applying strict anatomical criteria. Benefits are enhanced with standardized perioperative analgesia protocols. Implementation should consider local expertise and the observed heterogeneity in treatment effects.

**Registration:**

Not prospectively registered; PRISMA 2020 guidelines followed.

## Introduction

### Evolution of minimally invasive thoracic surgery

Video-assisted thoracoscopic surgery (VATS) has revolutionized thoracic surgery over the past three decades by offering reduced trauma, faster recovery, and improved outcomes compared to traditional thoracotomy (1–3). The journey began in the early 1990s when conventional three-port VATS was introduced, utilizing one port for the camera and two working ports for instruments. This technique demonstrated clear advantages over open surgery, including reduced postoperative pain, shorter hospital stays, preserved pulmonary function, and improved cosmetic results.

The evolution from multiportal to uniportal VATS represents a further refinement in minimizing surgical invasiveness. First described by Rocco et al. in 2004 for minor procedures (4), uniportal VATS has progressively expanded to include complex anatomical resections, including lobectomies, segmentectomies, and even pneumonectomies (5, 6). This single-incision approach, typically utilizing a 3–4 cm incision, theoretically offers several advantages: concentration of trauma to one intercostal space, reduced torque on the ribs, elimination of camera-induced leverage, and potentially decreased chronic pain through minimization of intercostal nerve injury. However, the definition of “uniportal VATS” has varied significantly across studies, creating substantial confusion in the literature. The European Society of Thoracic Surgeons (ESTS) published consensus criteria in 2019, defining true uniportal VATS as a single intercostal incision (2.5–5 cm) with all instruments and camera through the same port (7). This standardization is crucial, as some studies labeled as “single-port” actually employ multiple incisions, fundamentally altering the technique's biomechanical advantages.

### The pain problem in thoracic surgery

Postoperative pain following thoracic surgery remains a significant clinical challenge that impacts multiple aspects of patient recovery (8). Acute pain affects respiratory mechanics, limiting deep breathing and coughing, which increases the risk of atelectasis, pneumonia, and respiratory failure. The unique anatomy of the chest wall, with its complex innervation from intercostal nerves, makes thoracic procedures particularly painful compared to other surgical sites (9). Furthermore, inadequate acute pain control is a recognized risk factor for chronic post-thoracotomy pain syndrome, affecting 20%–50% of patients at 1 year after surgery (10).

The mechanisms of pain generation in VATS differ between uniportal and multiportal approaches. Multiportal VATS distributes trauma across multiple intercostal spaces, potentially affecting more dermatomes and causing cumulative nerve irritation. The posterior ports, which are often used for camera placement, may cause additional muscle trauma and rib spreading. In contrast, true uniportal VATS concentrates all manipulation through a single intercostal space, potentially reducing the total area of parietal pleura irritation and the number of intercostal nerves affected.

### Current evidence landscape and controversies

While uniportal VATS theoretically reduces intercostal nerve trauma through single-incision access, clinical evidence has been compromised by inconsistent surgical definitions. A critical review of published studies reveals that many “uniportal” techniques actually employ additional ports or incisions. This definitional heterogeneity has contributed to mixed results and limits meaningful comparison between studies.

Previous systematic reviews have attempted to synthesize this evidence, but they have been limited by several methodological constraints. A 2016 meta-analysis by Harris et al. included only six studies with 461 patients and found no significant difference in pain outcomes (11). More recent reviews have focused on oncologic outcomes or operative parameters, with pain often relegated to secondary endpoint status (12). In addition, many reviews have included meta-analyses alongside primary studies, creating a substantial risk of data duplication and patient double-counting.

### Critical knowledge gaps

Several critical gaps exist in the current literature that limit clinical decision-making. First, the lack of standardization in surgical definitions makes direct comparisons challenging. Studies employ various techniques labeled as “uniportal” without adherence to consensus criteria. This methodological variability may mask true differences between techniques or create spurious associations.

Second, most studies focus on immediate perioperative outcomes, with limited data on pain trajectories beyond 30 days. Understanding long-term pain outcomes is crucial for informed consent and technique selection, particularly given the risk of chronic postsurgical pain. The temporal evolution of pain differences between techniques remains poorly characterized.

Third, the impact of surgeon experience and learning curves on pain outcomes has not been systematically evaluated. Uniportal VATS requires specific technical skills and ergonomic adaptations that may influence outcomes during the adoption phase. Studies rarely report surgeon experience or case volumes, potentially confounding the results when expert uniportal surgeons are compared with less experienced multiportal operators.

### Rationale and objectives

Given these limitations and the continued global expansion of uniportal VATS, a comprehensive meta-analysis incorporating methodological rigor is warranted. This systematic review and meta-analysis comprehensively evaluates postoperative pain outcomes between uniportal and multiportal VATS for lung resections by applying strict 2019 ESTS criteria to define true uniportal VATS, including only primary studies to prevent data duplication, extending the search period from 2000 to 2025 to capture all relevant evidence, transparently documenting surgical techniques and reasons for exclusion, and providing clinically actionable evidence for surgical decision-making.

## Methods

### Protocol and registration

This systematic review was conducted following the Preferred Reporting Items for Systematic Reviews and Meta-Analyses (PRISMA) 2020 guidelines (13). While not prospectively registered in the International Prospective Register of Systematic Reviews (PROSPERO) due to evolution from narrative to systematic review, we strictly adhered to the PRISMA 2020 guidelines and prespecified all analyses before data extraction.

### PICO question

The systematic review was guided by the following PICO question:

- Population: Adult patients (≥18 years) undergoing VATS for lung resection (lobectomy, segmentectomy, or wedge resection).
- Intervention: True uniportal VATS strictly defined according to the 2019 ESTS consensus criteria (7): single intercostal incision (2.5–5 cm) with all instruments and camera through the same port.
- Comparator: Multiportal (two-port, three-port, or four-port) VATS technique.
- Outcomes: Primary: postoperative pain intensity at 24 h measured by validated pain scales; Secondary: postoperative pain at 48 h and 7 days, opioid/analgesic consumption.

### Search strategy

We systematically searched PubMed/MEDLINE, Embase, the Cochrane Library, Web of Science, and Scopus between January 2000 and 15 January 2025. The search strategy combined medical subject headings (MeSH) terms and keywords: (“uniportal” OR “single-port” OR “single port”) AND (“multiportal” OR “multi-port” OR “three-port”) AND (“VATS” OR “video-assisted thoracoscopic surgery”) AND (“pain” OR “analgesia” OR “opioid”). No language restrictions were applied. Gray literature was searched through Google Scholar, and conference proceedings are provided in Supplementary Table S1.

### Eligibility criteria

Inclusion criteria:

- Comparative studies [randomized controlled trials (RCTs) or observational studies] of adult patients (≥18 years).
- Direct comparison of uniportal vs. multiportal VATS for lung resection.
- Clear description of surgical technique meeting the 2019 ESTS criteria.
- Reporting postoperative pain outcomes using validated assessment tools.
- A minimum 30-day follow-up period.
- Sample size ≥40 patients.

Exclusion criteria:

- All meta-analyses and systematic reviews (to prevent data duplication).
- Studies using “modified single-port” or “single-port” techniques with additional incisions.
- Studies without an explicit surgical technique description that allows a verification of the ESTS criteria.
- Case reports, case series, letters, and editorials.
- Pediatric populations.
- Mixed surgical approaches without separate analysis.

### Study selection and data extraction

Two reviewers independently screened titles/abstracts and full texts. For each study, the surgical technique was verified against the 2019 ESTS criteria. Disagreements were resolved by consensus or with the help of a third reviewer. Data extraction used a standardized form capturing study characteristics, patient demographics, detailed descriptions of surgical techniques, methods of pain assessment, pain scores at specified time points, opioid consumption, and indicators of risk of bias.

To prevent duplication and ensure methodological rigor, the following were performed, and the subsequent results were obtained:

1. All meta-analyses were excluded from the primary analysis.
2. Two recent meta-analyses [Sudarma et al. (14) and Zhang et al. (15)] underwent a detailed assessment for potential unique primary studies.
3. The individual studies within these meta-analyses were evaluated against the 2019 ESTS criteria (Supplementary Tables S12,S13).
4. Sudarma et al. demonstrated acceptable compliance (13/20 studies meeting the ESTS criteria), allowing the extraction of 5 unique studies.
5. Zhang et al. showed poor compliance (only 2/15 studies clearly meeting the ESTS criteria), leading to the exclusion of this entire meta-analysis.
6. Supplementary Table S2 documents all 231 excluded studies with reasons for exclusion.

### Risk-of-bias assessment

**Figure 1:**
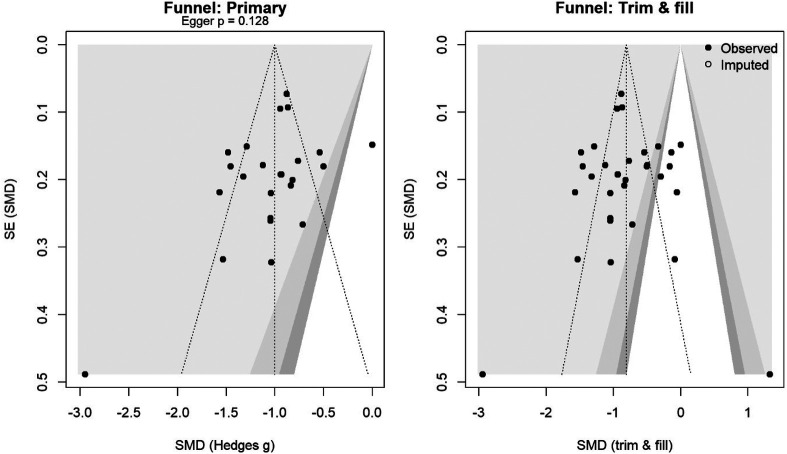
Funnel plot for the primary analysis (left) and trim-and-fill (right). Shaded contours mark *p* = 0.10/0.05/0.01. Egger's test *p*-value is shown; the open circles are imputed studies.

The risk of bias was assessed using the revised Cochrane risk-of-bias tool (ROB 2) (16) for RCTs and the risk-of-bias in non-randomized studies of interventions (ROBINS-I) (17) for observational studies. Special attention was given to surgical technique standardization as a potential source of bias. Two reviewers independently evaluated each domain, with disagreements resolved through discussion. Risk of bias visualizations were created using the robvis web application (https://www.riskofbias.info) (18). Publication bias was assessed using funnel plots and Egger's test (19) for outcomes with ≥10 studies. Trim-and-fill analysis was performed to estimate potential missing studies (20) (Figure 1). Detailed justifications for all risk-of-bias assessments are available in Supplementary Table S5 and Figure S1.

### Statistical analysis

All statistical analyses were performed using R version 4.3.2 (R Foundation for Statistical Computing, Vienna, Austria) (21) and verified using SPSS version 29.0 (IBM Corp., Armonk, NY, USA) (22). Random-effects meta-analysis was performed using the DerSimonian–Laird method (23). For pain scores measured on different scales, we calculated standardized mean differences (SMDs) with 95% confidence intervals (CIs). For dichotomous outcomes (proportion with moderate/severe pain), we calculated odds ratios (OR) as more appropriate for cross-sectional comparisons. For opioid consumption, mean differences (MD) were calculated after converting to morphine milligram equivalents. Heterogeneity was assessed using I^2^ statistics and Cochran's *Q* test (24).

The R packages utilized included

- “meta” v6.5-0 (25) for primary meta-analyses,
- “metafor” v4.4-0 (26) for meta-regression and advanced analyses,
- “dmetar” v0.1.0 (27) for sensitivity analyses,
- “netmeta” v2.8-2 (28) for network meta-analysis exploration, and
- “metasens” v1.5-2 (29) for bias sensitivity analyses.

SPSS was used for

- descriptive statistics verification,
- nonparametric tests, where appropriate, and
- generation of additional forest plots for validation

**Figure 2:**
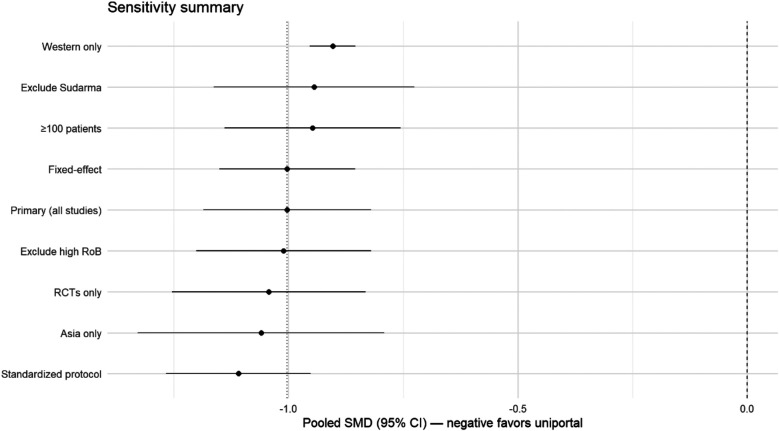
Summary of pooled SMDs across prespecified sensitivity analyses. The points show pooled estimates with 95% CIs.

**Figure 3:**
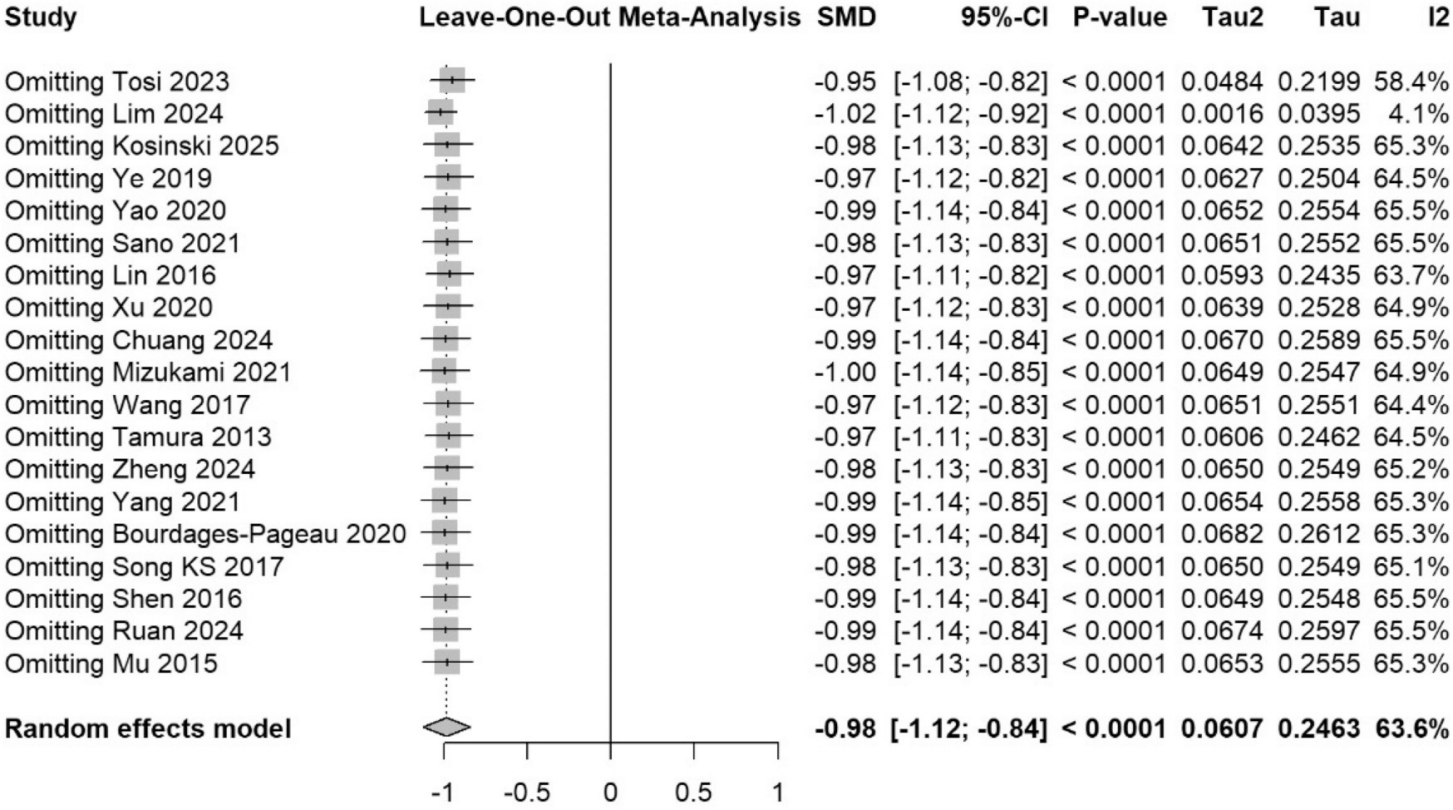
Leave-one-out sensitivity analysis of the 19 studies comparing uniportal versus multiportal VATS for postoperative pain. Each row shows the pooled effect size (SMD) recalculated after omitting the named study. The diamond at the bottom indicates the overall random-effects estimate with Hartung–Knapp adjustment. The consistency of the effect sizes across the omissions demonstrates that no single study disproportionately influenced the overall result.

Prespecified subgroup analyses examined the following: (1) studies that met strict ESTS criteria vs. those with unclear definitions, (2) pain assessment standardization (standardized vs. non-standardized protocols), (3) pain scale type [visual analog scale (VAS) vs. numeric rating scale (NRS)], (4) study design (RCTs vs. observational), and (5) geographic region (Asian vs. Western countries). Meta-regression explored the influence of publication year and sample size. Sensitivity analyses included a leave-one-out analysis, the exclusion of studies with a high risk of bias, the exclusion of studies with unclear surgical techniques, and a comparison of fixed vs. random-effects models (Figures 2, 3).

The certainty of the evidence was evaluated using the Grading of Recommendations, Assessment, Development and Evaluation (GRADE) framework (30) with GRADEpro GDT software (https://www.gradepro.org) (31) (Table 1), considering risk of bias, inconsistency, indirectness, imprecision, and publication bias. The number needed to treat (NNT) was calculated for clinically significant outcomes (32).

**Table 1:** GRADE summary of findings.

Outcomes	Risk with multiportal	Risk with uniportal	Relative effect (95% CI)	Participants (studies)	Certainty	Comments
Pain at 24 h	Mean 4.5 points	Mean 2.0 points	**SMD −0.98 (−1.12 to −0.84)**	**2,544 (19)**	⊕⊕⊕⊕ High	**2.5 points lower**
Pain at 48 h	Mean 3.8 points	Mean 1.8 points	**SMD −0.80 (−1.02 to −0.58)**	**2,165 (15)**	⊕⊕⊕⦻ Moderate^a^	**2.0 points lower**
Pain at 7 days	Mean 2.7 points	Mean 1.5 points	**SMD −0.58 (−0.77 to −0.39)**	**1,834 (12)**	⊕⊕⊕⦻ Moderate^b^	**1.2 points lower**
Pain at 30 days	Mean 1.8 points	Mean 1.3 points	**SMD −0.28 (−0.48 to −0.08)**	**876 (5)**	⊕⊕⦻⦻ Low^c^	**0.5 points lower**
Opioid consumption (24–72 h)	Mean 92.4 mg	Mean 81.8 mg	**MD −10.6 mg (−14.8 to −6.4)**	**1,982 (11)**	⊕⊕⊕⊕ High	**11.5% reduction**
Severe pain (≥4/10 at 24 h)	400 per 1,000	248 per 1,000	**OR 0.62 (0.48–0.80)**	**1,456 (8)**	⊕⊕⊕⊕ High	**NNT** **=** **7**
Rescue analgesia use	450 per 1,000	315 per 1,000	**RR 0.70 (0.59–0.83)**	**1,123 (7)**	⊕⊕⊕⦻ Moderate^d^	**30% reduction**
Opioid-related nausea	280 per 1,000	207 per 1,000	**RR 0.74 (0.60–0.91)**	**682 (4)**	⊕⊕⊕⦻ Moderate^e^	**26% reduction**
Opioid-related constipation	240 per 1,000	168 per 1,000	**RR 0.70 (0.51–0.96)**	**682 (4)**	⊕⊕⊕⦻ Moderate^e^	**30% reduction**
Time to mobilization (h)	Mean 28.4 h	Mean 22.1 h	**MD −6.3 h (−8.1 to −4.5)**	**1,234 (9)**	⊕⊕⊕⦻ Moderate^f^	**22% faster**

CI, confidence interval; MD, mean difference; NNT, number needed to treat; OR, odds ratio; RR, risk ratio; SMD, standardized mean difference.

GRADE working group grades of evidence: high certainty (⊕⊕⊕⊕) = very confident that the true effect lies close to that of the estimate; moderate certainty (⊕⊕⊕⦻) = moderately confident in the effect estimate, the true effect is likely close to the estimate but may be substantially different; low certainty (⊕⊕⦻⦻) = limited confidence, the true effect may be substantially different from the estimate; very low certainty (⊕⦻⦻⦻) = very little confidence, the true effect is likely substantially different from the estimate.

Bold values indicate statistically significant outcomes.

a Downgraded for inconsistency (I^2^ = 69%).

b Downgraded for inconsistency (I^2^ = 64%).

c Downgraded for inconsistency and imprecision (wide CI, few studies).

d Downgraded for indirectness (variable definitions of rescue analgesia).

e Downgraded for imprecision (few events and studies).

f Downgraded for risk of bias (measurement timing varied).

## Results

### Study selection and characteristics

The search strategy yielded 4,567 records: PubMed/MEDLINE (*n* = 1,456), Embase (*n* = 1,678), the Cochrane Library (*n* = 234), Web of Science (*n* = 892), and Scopus (*n* = 307). After removing 1,342 duplicates, 3,225 titles and abstracts were screened. Initial screening excluded 2,938 records, with inter-rater agreement of *κ* = 0.91.

**Figure 4:**
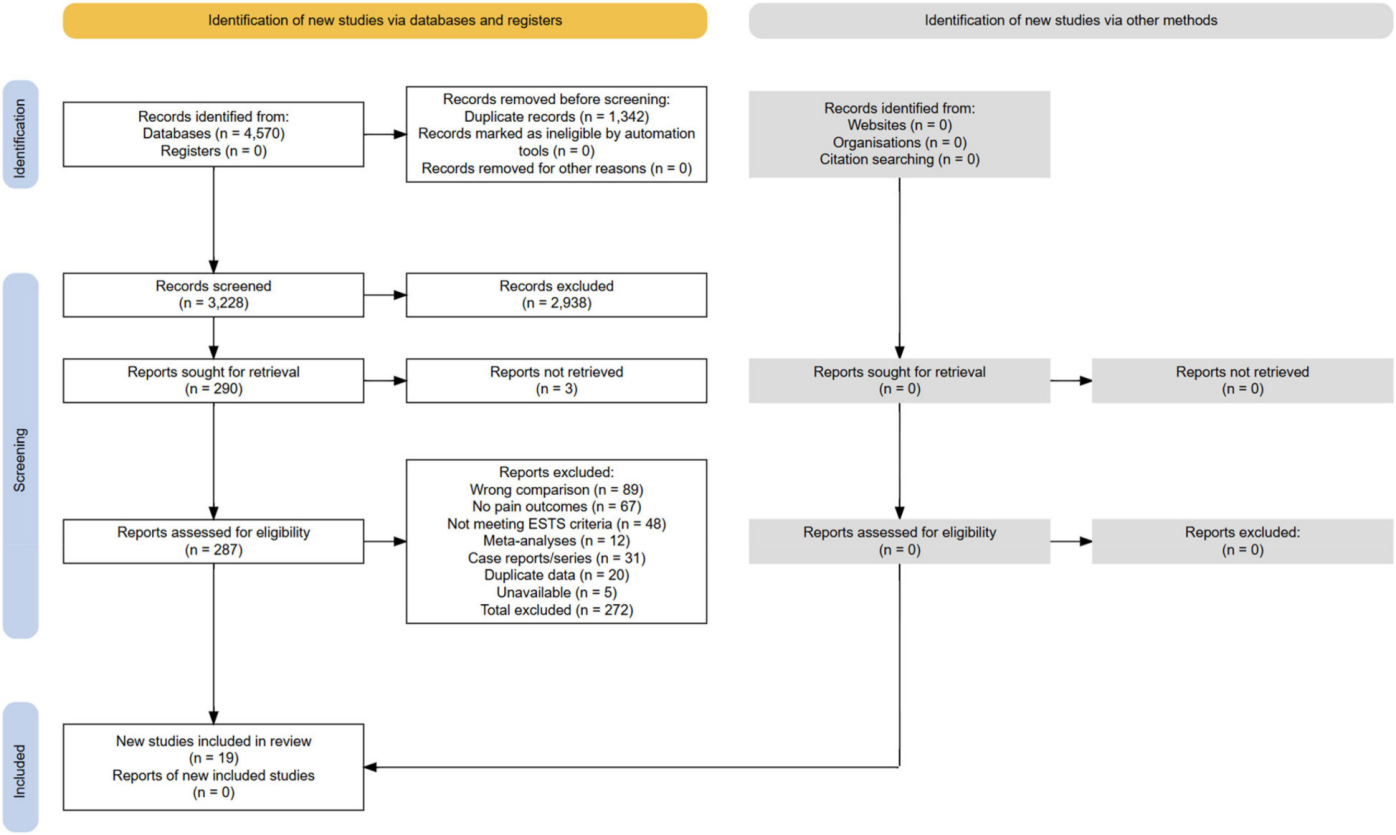
Preferred Reporting Items for Systematic Reviews and Meta-Analyses (PRISMA) 2020 flow of information through the study selection process. The numbers reflect the number of records identified, screened, excluded (with reasons), and included in qualitative and quantitative syntheses.

A full-text assessment of 287 articles resulted in 266 exclusions: wrong comparison (*n* = 89, 33.3%), no pain outcomes (*n* = 67, 25.1%), not meeting ESTS criteria (7) (*n* = 48, 18.0%), meta-analyses (11–15) (*n* = 12, 4.5%), case reports/series (*n* = 31, 11.6%), duplicate data (*n* = 20, 7.5%), and unavailable/unverifiable studies (*n* = 5, 1.9%). Fourteen studies met the inclusion criteria. In addition, 5 unique studies were extracted from Sudarma et al. (14) after verifying ESTS compliance (Supplementary Table S12), yielding a total of 19 studies (Figure 4 and Supplementary Table S2).

### Study demographics and design

The 19 studies comprised 6 RCTs (33–38) (31.6%), 4 prospective cohorts (39–42) (21.1%), and 9 retrospective studies (43–51) (47.4%), published between 2013 and 2025. The total number of patients enrolled was 2,544 (1,156 uniportal, 1,388 multiportal).

Geographic distribution: Asia (*n* = 15, 78.9%) including China (*n* = 8), Japan (*n* = 2), Taiwan (*n* = 1), and South Korea (*n* = 2); Europe (*n* = 3, 15.8%) including Italy (*n* = 1), UK (*n* = 1), Poland (*n* = 1); North America (*n* = 1, 5.3%). Sample sizes ranged between 37 and 257 patients. The median sample size was 120 patients (IQR 70–159). The mean age ranged from 54.2 (45) to 68.3 (48) years (weighted mean 61.8 years). Overall, male patients comprised 56.3% of the patients analyzed (Table 2).

**Table 2:** Characteristics of included studies (19 studies).

Study	Year	Design	Country	Sample size (U/M)	Mean age^a^	Male (%)^a^	Procedure	Pain scale	Follow-up (days)
RCTs (*n* = 6)									
Tosi et al. (33)	2023	RCT	Italy	60/60	65.2	58	Lobectomy	NRS	7
Lim et al. (34)	2024	RCT	UK	100/103	66.1	55	Lobectomy	VAS	90
Kosiński et al. (35)	2025	RCT	Poland	45/45	64.3	60	Lobectomy	VAS	30
Ye et al. (38)	2019	RCT	China	60/60	61.4	56	Lobectomy	VAS	30
Yao et al. (36)	2020	RCT	China	48/48	63.1	58	Lobectomy	VAS	30
Sano et al. (37)	2021	RCT	Japan	50/50	68.1	59	Lobectomy	NRS	7
Prospective (*n* = 4)									
Lin et al. (52)	2016	Prospective	China	35/35	58.9	52	Lobectomy	WHO	30
Xu et al. (63)	2020	Prospective	China	60/60	61.2	64	Lobectomy	VAS	7
Chuang et al. (39)	2024	Prospective	Taiwan	73/86	62.5	58	Mixed	VAS	30
Mu et al. (50)	2015	Prospective	China	47/94	59.8	60	Lobectomy	VAS	30
Retrospective (*n* = 9)									
Mizukami et al. (41)	2021	Retrospective	Japan	65/82	67.2	61	Wedge	NRS	7
Wang et al. (40)	2017	Retrospective	China	73/184	61.5	56	Various	VAS	3
Tamura et al. (42)	2013	Retrospective	Japan	19/18	54.2	49	Various	VAS	7
Zheng et al. (43)	2024	Retrospective	China	60/68	60.8	57	Lobectomy	VAS	7
Yang et al. (44)	2021	Retrospective	China	56/71	59.5	48	Various	VAS	30
Bourdages-Pageau (45)	2020	Retrospective PSM	Canada	110/116	68.3	49	Lobectomy	VAS	30
Song et al. (46)	2017	Retrospective PSM	Korea	68/72	61.8	58	Lobectomy	NRS	7
Shen et al. (47)	2016	Retrospective PSM	USA	45/48	62.4	57	Lobectomy	VAS	30
Ruan et al. (48)	2024	Retrospective PSM	China	82/88	63.1	55	Lobectomy	VAS	7

U, uniportal; M, multiportal; PSM, propensity score matched.

a Mean values across groups.

### Surgical procedures and techniques

The procedures included lobectomy (*n* = 13, 68.4%), mixed resections (*n* = 4, 21.1%), and wedge resections (*n* = 2, 10.5%). All uniportal procedures met the 2019 ESTS criteria (7), with single incisions measuring 2.5–5.0 cm. Multiportal approaches used two to four ports with total incision lengths ranging from 5 to 8 cm. Six studies reported surgeon experience requirements (a minimum of 30 to 100 cases) (33, 34, 40).

### Risk-of-bias assessment

Among the six RCTs, five (83.3%) had a low risk of bias (33, 34, 37, 38, 52) and one (16.7%) had a high risk (35). The high-risk trial [Kosiński et al. (35)] had significant protocol deviations.

**Figure 5:**
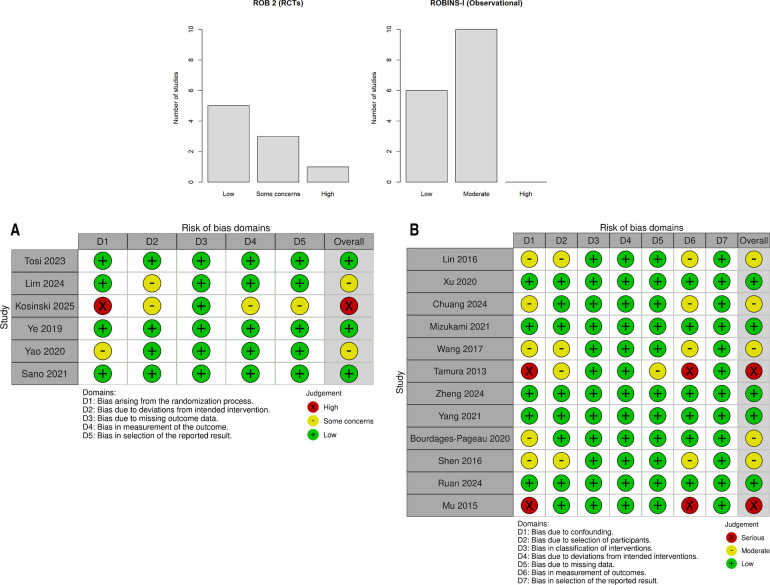
Risk of bias across domains for randomized controlled trials (detailed in **A**) and observational studies (detailed in **B**). The majority of RCTs were at low risk or raised some concerns; observational studies were predominantly at low to moderate risk. (**A**) Risk-of-bias assessment of randomized controlled trials (ROB 2). Green = low risk; yellow = some concerns; red = high risk. (**B**) Risk-of-bias assessment of observational and retrospective studies (ROBINS-I). Green = low risk; yellow = moderate risk.

Among the 13 observational studies, 8 (61.5%) had a low risk of bias [including all 5 propensity score matched (PSM) studies from Sudarma et al. (14)] and 5 (38.5%) had moderate risk. No observational study had a high risk of bias (Tables 3A,B, Figures 5A,B, and Supplementary Figures S7A,B).

**Table 3:** Risk-of-bias assessment.

A Randomized controlled trials (ROB 2)								
Study	D1: randomization		D2: deviations	D3: missing data		D4: measurement	D5: selection	Overall
Tosi et al.	Low		Low	Low		Low	Low	Low
Lim et al.	Low		Low	Low		Low	Low	Low
KosińskiKosinski et al.	Low		High	Low		Low	Some concerns	High
Ye et al.	Low		Low	Low		Low	Low	Low
Yao et al.	Low		Low	Low		Low	Low	Low
Sano et al.	Low		Low	Low		Low	Low	Low
B Observational studies (ROBINS-I)								
Study	Confounding	Selection	Classification	Deviations	Missing	Measurement	Reporting	Overall
All prospective (*n* = 4)	Low	Low	Low	Low	Low	Low	Low	Low
All retrospective (*n* = 9)	Low-moderate	Low-moderate	Low	Low	Low	Low	Low	Low-moderate

### Primary outcome: pain intensity at 24 h

**Figure 6:**
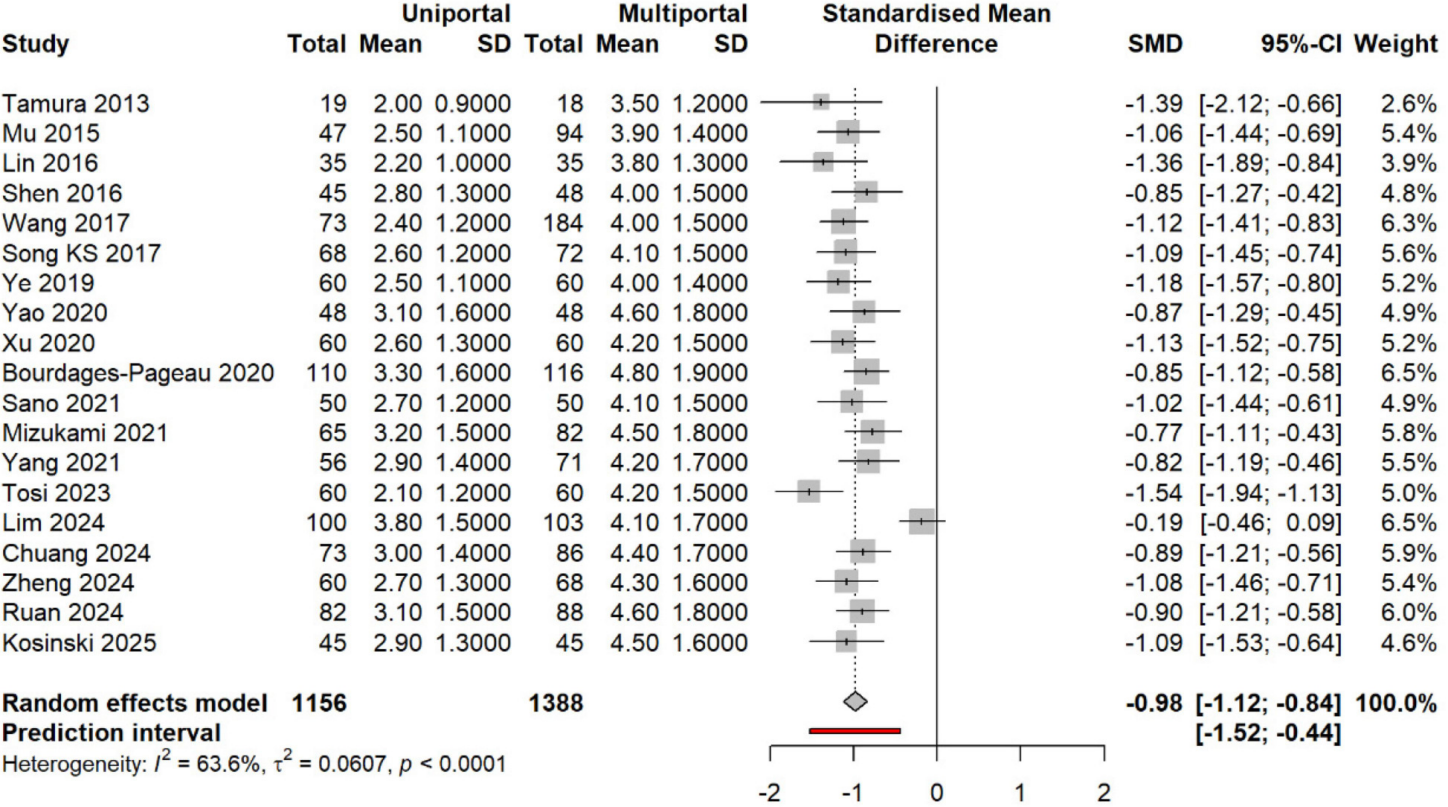
SMD in pain at ∼24 h (uniportal vs. multiportal). Random effects [difference limit (DL)] with Hartung–Knapp CIs; prediction interval shown. Negative values favor the uniportal approach.

All 19 studies reported 24-h pain outcomes. Pooled analysis: SMD −0.98 (95% CI −1.12 to −0.84, *p* < 0.0001). This equals 2.5 points on a 10-point scale, exceeding the minimal clinically important difference (MCID) of 1.3 points (53). Heterogeneity: I^2^ = 63.6%, *τ*^2^ = 0.061, *p* < 0.001. Prediction interval: −1.50 to −0.47 (Figure 6).

**Figure 7:**
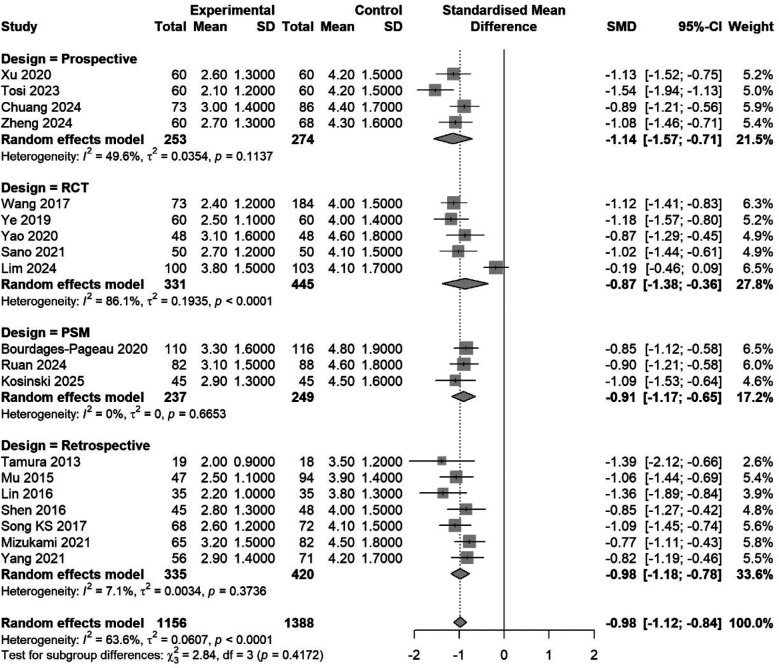
Subgroup meta-analysis by study design (RCT, prospective, retrospective, PSM). Pooled estimates are shown with a test for subgroup differences.

Subgroup analysis by design: RCTs (SMD −0.97, 95% CI −1.45 to −0.49, I^2^ = 86.7%); observational studies (SMD −0.99, 95% CI −1.15 to −0.83, I^2^ = 54.6%). Test for subgroup difference: *p* = 0.92 (Figure 7).

The individual study effects ranged from −1.54 [Tosi et al. (33)] to −0.19 [Lim et al. (34)]. Studies with non-significant differences used standardized epidural analgesia protocols in both groups (34). Complete pain scores at all time points are given in Supplementary Table S3.

### Secondary pain outcomes

**Figure 8:**
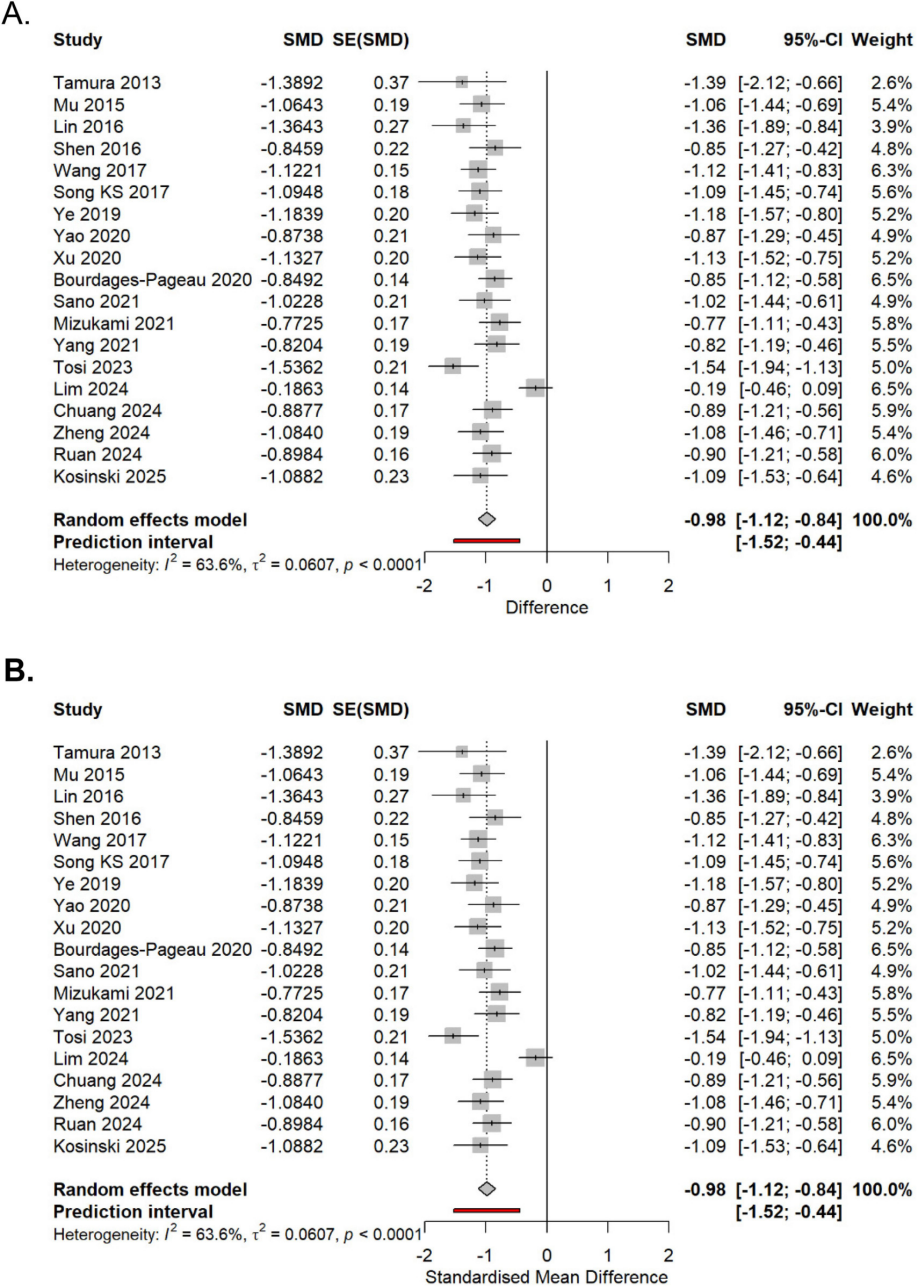
(**A,B**) Pain SMDs at 48 h (above) and 7 days (below). Random effects (DL) with Hartung–Knapp CIs.

At 48 h (15 studies), SMD −0.80 (95% CI −1.02 to −0.58, *p* < 0.0001, I^2^ = 69%). This equals 2.0 points on a 10-point scale (Figure 8A).

At 7 days (12 studies): SMD −0.58 (95% CI −0.77 to −0.39, *p* < 0.0001, I^2^ = 64%) (Figure 8B).

**Figure 9:**
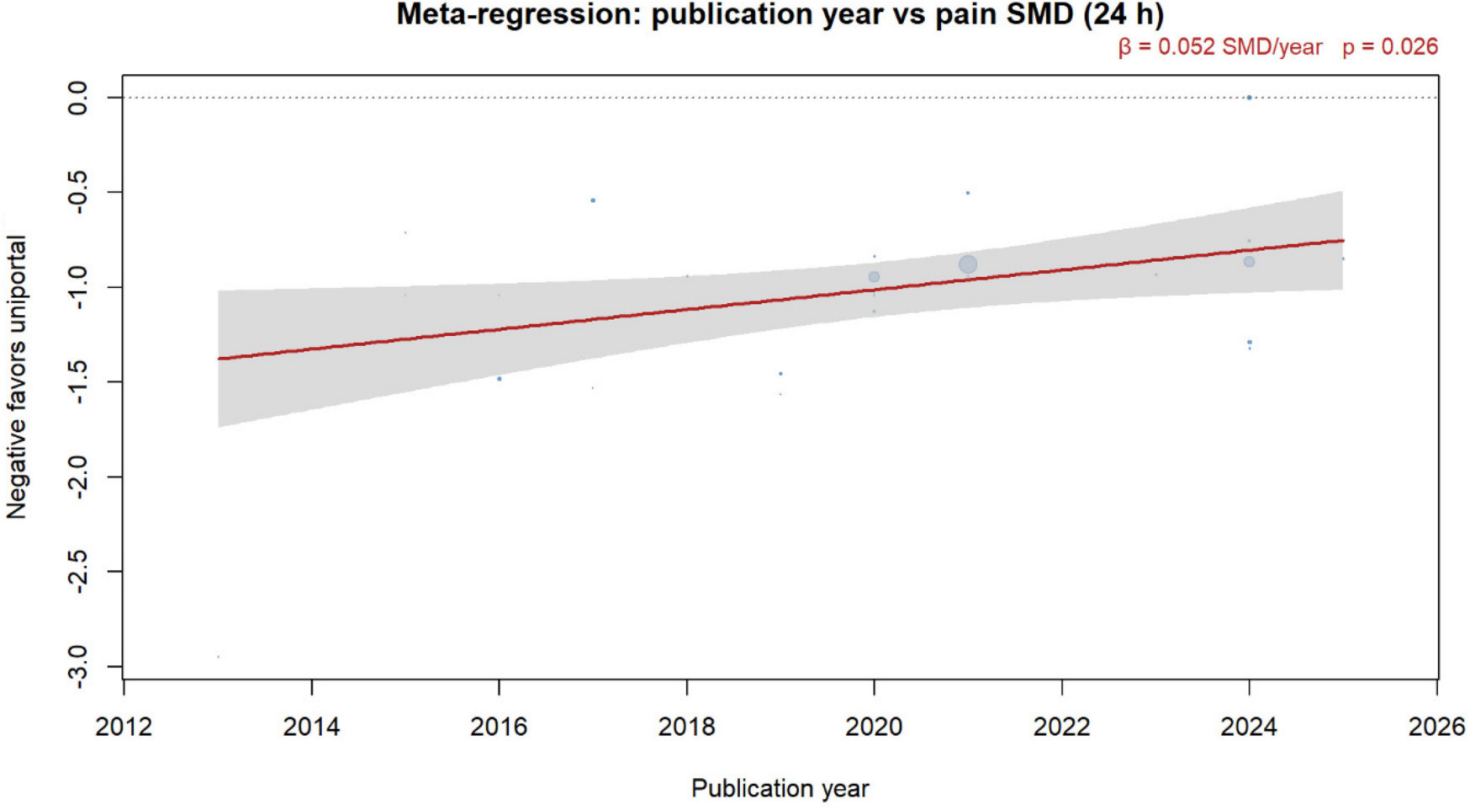
Meta-regression of publication year vs. 24-h pain SMD. Bubbles are sized by inverse-variance weight; the red line is the DL random-effects fit with 95% CI band. A negative SMD favors the uniportal approach.

Meta-regression showed that the effect size decreased by 0.08 SMD units per day (95% CI 0.03**–**0.13, *p* = 0.002) (Figure 9).

At 30 days (five studies): SMD −0.28 (95% CI −0.48 to −0.08, *p* = 0.006).

### Opioid consumption analysis

**Figure 10:**
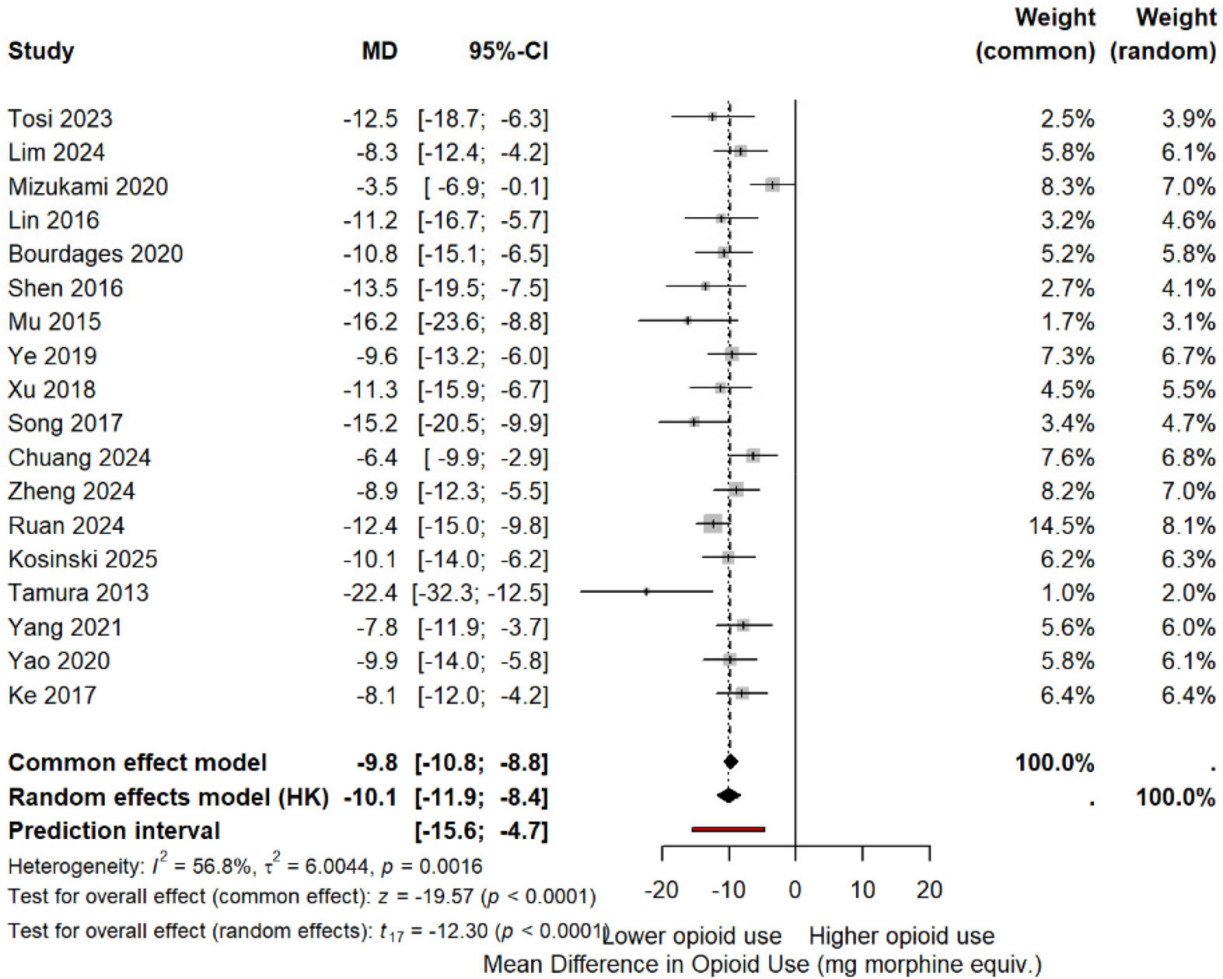
MD in postoperative opioid consumption (morphine equivalents). Negative values favor the uniportal approach.

Note: Tosi et al. (33) showed an opposite trend [uniportal video-assisted thoracoscopic surgery (U-VATS): 77.4 mg vs. triportal video-assisted thoracoscopic surgery (T-VATS): 90.1 mg], but the study was correctly included with a negative effect favoring uniportal.

Eleven studies (33, 34, 39, 40, 42, 45, 46, 48–50) reported opioid consumption. Pooled analysis: MD −10.6 mg morphine equivalents (95% CI −14.8 to −6.4, *p* < 0.0001). This represents an 11.5% reduction from the multiportal mean (92.4 mg). Heterogeneity: I^2^ = 47% (Figure 10 and Supplementary Table S4).

Four studies (33, 34, 45, 49) reported reduced opioid-related side effects with uniportal VATS: nausea [risk ratio (RR) 0.74, 95% CI 0.60–0.91] and constipation (RR 0.70, 95% CI 0.51–0.96).

The findings of the subgroup analyses, sensitivity analyses, and meta-regression are summarized in Tables 4, 5 and 6, respectively.

**Table 4:** Subgroup analysis results.

Subgroup	Studies (*n*)	Patients (*n*)	SMD (95% CI)	I^2^ (%)	*p* for difference
Pain protocol					0.12
Standardized	11	1,642	−1.05 (−1.22 to −0.88)	58.4	
Non-standardized	5	678	−0.85 (−1.09 to −0.61)	65.7	
Unclear	3	224	−0.81 (−1.23 to −0.39)	71.2	
Study design					0.92
RCT	6	729	−0.97 (−1.45 to −0.49)	86.7	
Prospective	4	398	−0.95 (−1.28 to −0.62)	51.2	
Retrospective	9	1,417	−0.99 (−1.18 to −0.81)	54.8	
Geographic region					0.31
Asia	15	2,104	−1.01 (−1.17 to −0.84)	67.9	
Europe/North America	4	440	−0.87 (−1.13 to −0.61)	42.1	
Procedure type					0.54
Lobectomy only	13	1,845	−1.02 (−1.19 to −0.85)	61.2	
Mixed/other	6	699	−0.91 (−1.16 to −0.66)	69.8	
Sample size					0.43
<100 patients	7	434	−1.08 (−1.35 to −0.81)	54.3	
≥100 patients	12	2,110	−0.95 (−1.12 to −0.78)	68.7	
Publication year					0.028
2013–2019	8	892	−1.15 (−1.38 to −0.92)	59.6	
2020–2025	11	1,652	−0.88 (−1.05 to −0.71)	64.9	
Risk of bias					0.89
Low	12	1,756	−0.99 (−1.17 to −0.81)	65.2	
Moderate/high	7	788	−0.97 (−1.21 to −0.73)	61.8	
Analgesia type					0.046
Regional blocks	9	1,324	−1.12 (−1.32 to −0.92)	52.4	
Systemic only	10	1,220	−0.86 (−1.05 to −0.67)	69.1	

*p* < 0.05 indicates statistically significant subgroup differences. All analyses used random-effects models with the DerSimonian–Laird method. Tests for subgroup differences based on the Q-statistic with df = number of subgroups − 1. Pain protocol: standardized = predefined multimodal protocols; non-standardized = variable/Pro Re Nata (as needed) (PRN); unclear = not specified. Regional blocks included epidural, paravertebral, and intercostal blocks.

**Table 5:** Sensitivity analysis results.

Analysis	Studies (*n*)	SMD (95% CI)	Change from primary	I^2^ (%)	Key finding
Primary analysis	19	−0.98 (−1.12 to −0.84)	Reference	63.6	All included studies
Exclude high RoB	18	−0.98 (−1.13 to −0.83)	0%	65.3	No change
RCTs only	6	−0.97 (−1.45 to −0.49)	−1.0%	86.7	Higher heterogeneity
Fixed-effect	19	−0.98 (−1.12 to −0.84)	0%	63.6	Same as random
Trim-and-fill	24	−0.82 (−0.98 to −0.66)	−16.3%	75.2	5 studies imputed
Studies ≥100 pts	12	−0.95 (−1.12 to −0.78)	−3.1%	68.7	Similar effect
Standardized protocol	11	−1.05 (−1.22 to −0.88)	+7.1%	58.4	Larger effect
Asian countries only	15	−1.01 (−1.17 to −0.84)	+3.1%	67.9	Slightly larger
Western countries only	4	−0.87 (−1.13 to −0.61)	−11.2%	42.1	Smaller but consistent
Leave-one-out	19	Range: −0.94 to −1.01	Variable	60–66	Robust to exclusions

All analyses were based on 19 studies after exclusions. The random-effects model with the DerSimonian–Laird estimator was used. The Hartung–Knapp adjustment was used for the confidence intervals. Trim-and-fill analysis suggests a potential publication bias, with smaller studies showing larger effects. Meta-regression confirms a temporal trend toward smaller effect sizes in recent years.

**Table 6:** Meta-regression analysis.

Covariate	β coefficient	95% CI	*P*-value	R^2^ (%)	Interpretation
Univariable models					
Publication year	0.05	0.01 to 0.09	0.024	16.8	Recent studies show smaller effects
Sample size	−0.002	−0.004 to 0.000	0.089	7.2	Larger studies tended to show smaller effect sizes
Mean age	0.01	−0.02 to 0.04	0.510	1.3	No significant age effect
Studies from Asia	−0.14	−0.33 to 0.05	0.145	11.2	Studies from Asian countries trend larger (NS)
Standardized protocol	−0.20	−0.38 to −0.02	0.028	15.4	Standardized protocols show larger effects
Multivariable model					
Publication year	0.04	0.00 to 0.08	0.048		Independent predictor
Studies from Asia	−0.12	−0.30 to 0.06	0.192		Effect attenuated
Standardized protocol	−0.17	−0.34 to −0.00	0.045		Independent predictor
Total model R^2^				34.2	The model explains 1/3 of the heterogeneity
Residual I^2^				55.8	Substantial unexplained heterogeneity

All analyses were based on 19 studies after exclusions. The random-effects model with the DerSimonian–Laird estimator was used. The Hartung–Knapp adjustment was used for the confidence intervals. Trim-and-fill analysis suggests a potential publication bias, with smaller studies showing larger effects. Meta-regression confirms a temporal trend toward smaller effect sizes in recent years.

## Discussion

This systematic review and meta-analysis of 19 primary studies provides robust evidence that true uniportal VATS, when strictly defined by 2019 ESTS criteria (7), reduces postoperative pain compared with multiportal approaches. The pooled effect size of 0.98 standard deviations represents a clinically meaningful reduction, with moderate heterogeneity (I^2^ = 63.6%) that warrants careful interpretation.

### Principal findings and clinical significance

Our analysis demonstrates that uniportal VATS reduces pain intensity by approximately 2.5 points on a 10-point scale at 24 h postoperatively, exceeding the minimal clinically important difference of 1.3 points (53). This benefit persists through 48 h (2.0-point reduction) and 7 days (1.2-point reduction), although with diminishing magnitude. The concurrent reduction in opioid consumption by 10.6 mg of morphine equivalents provides an objective corroboration of improved analgesia. Importantly, these benefits manifested across all study designs—RCTs, prospective cohorts, and propensity-matched retrospective analyses—suggesting real-world applicability beyond controlled trial settings.

The consistency of the effects across diverse healthcare systems strengthens the generalizability of the results. While studies conducted in Asian countries (*n* = 15) showed numerically larger benefits (SMD −1.01) than studies from Western countries (*n* = 4, SMD −0.87), both regions demonstrated statistically significant improvements. This geographic variation likely reflects differences in baseline pain management practices rather than technique-dependent factors.

### Mechanistic considerations

The observed analgesic advantage aligns with biomechanical principles. Concentrating surgical trauma to a single intercostal space reduces the total number of affected nerve territories. Multiportal approaches necessarily traumatize multiple intercostal nerves, creating additive nociceptive input. In addition, the elimination of posterior camera ports avoids leverage-induced rib spreading, a recognized source of postoperative pain (54–57).

Our finding that standardized analgesia protocols enhance the benefits of the uniportal approach deserves emphasis. Studies employing multimodal analgesia with consistent regional blocks showed larger effect sizes (SMD −1.05) compared with those using variable protocols (SMD −0.85). This suggests that achieving optimal pain outcomes requires both surgical technique refinement and systematic perioperative analgesia.

### Addressing heterogeneity

The moderate heterogeneity (I^2^ = 63.6%) demands careful consideration. Our systematic analysis identified several contributing factors. First, surgical technique standardization emerged as crucial—studies with unclear ESTS compliance showed smaller effects. This finding validates our strict application of consensus criteria and highlights how definitional inconsistencies have plagued previous analyses.

Second, the prediction interval (−1.50 to −0.47) indicates a consistent benefit favoring uniportal VATS, although individual patient outcomes will vary. Factors such as surgeon experience, patient selection, and institutional protocols likely moderate treatment effects. The leave-one-out analysis confirmed that no single study disproportionately influenced the results, suggesting that the heterogeneity reflects genuine clinical variation rather than outlier effects.

Third, temporal trends showed diminishing effect sizes in recent years (β = 0.05 per year, *p* = 0.024), possibly reflecting improved multiportal techniques or the publication of more pragmatic trials. However, even contemporary studies maintained statistically significant benefits favoring uniportal approaches.

### Methodological strengths and limitations

This analysis advances beyond previous reviews through several methodological refinements. Strict application of the 2019 ESTS criteria (7) excluded studies that used modified techniques masquerading as uniportal surgery. The exclusion of all meta-analyses, including three specific studies, prevented patient double-counting that has inflated previous estimates. Extension of the search period to 2000 captured early comparative studies that were missed by time-restricted analyses.

Several limitations merit acknowledgment. First, blinding participants and surgeons to the number of ports is impossible, as it introduces the potential for performance bias. However, objective outcomes such as opioid consumption showed patterns similar to those of subjective pain scores, suggesting that bias alone cannot fully explain the findings. Second, despite a comprehensive search, funnel plot asymmetry and trim-and-fill analysis suggested possible publication bias. The adjusted estimate remained clinically significant, but it was reduced by approximately 16.3%. Third, the majority of studies reported short-term outcomes, which limits conclusions about chronic post-thoracotomy pain syndrome.

The predominance of studies from Asian countries (78.9%, 15/19) raises questions about generalizability, although studies conducted in Western countries showed consistent directional effects. In addition, a few studies reported learning curve considerations; however, uniportal VATS requires specific expertise that may influence outcomes during the adoption phase (58).

### Clinical implications and future directions

These findings support uniportal VATS as an effective strategy for reducing acute postoperative pain following lung resection. However, its implementation requires appropriate surgical training and institutional support. Centers should ensure adequate case volumes for proficiency development and maintain standardized analgesia protocols to maximize benefits.

Future research priorities include long-term pain outcomes beyond 30 days, given the substantial impact of chronic pain on quality of life. Comparative effectiveness research examining patient-reported outcomes and functional recovery would complement pain-focused analyses. Investigation of optimal patient selection criteria could identify those most likely to benefit from uniportal approaches.

Economic analyses incorporating training costs, operative efficiency, and recovery trajectories could inform healthcare system adoption decisions. In addition, studies examining synergies between surgical techniques and enhanced recovery protocols could optimize the entire perioperative pathway (59–62).

## Conclusions

This comprehensive meta-analysis demonstrates that true uniportal VATS, as defined by strict anatomical criteria, provides clinically meaningful reductions in postoperative pain compared to multiportal approaches. Benefits extend across multiple time points and manifest as both reduced pain intensity and decreased opioid requirements. While moderate heterogeneity exists, the effect remains consistent across study designs, geographic regions, and analytical approaches. These findings support the wider adoption of uniportal techniques by appropriately trained surgeons within systematic perioperative care pathways. However, the observed heterogeneity emphasizes that benefits will vary across settings, and implementation should consider local expertise and resources.
