# Supplementary material for: Postoperative pain outcomes following uniportal vs. multiportal video-assisted thoracoscopic surgery: a systematic review and meta-analysis
Source: Front Surg. 2025 Nov 10;12:1689456. doi: 10.3389/fsurg.2025.1689456 (PMC12641003; doi:10.3389/fsurg.2025.1689456)
Supplement: Supplementary file 1 [file Datasheet1.pdf]

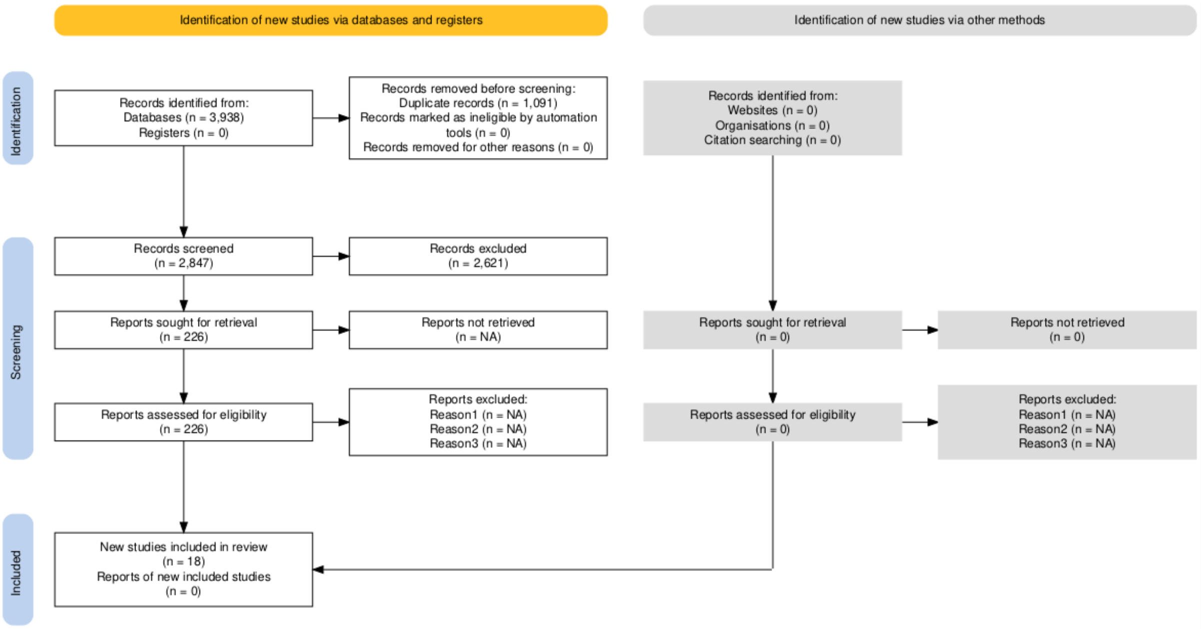

Supplementary Figure S1. PRISMA 2020 Flow Diagram*

PRISMA 2020 Flow Diagram

Legend: Study selection process following PRISMA 2020 guidelines showing the flow of information through different phases of the systematic review. Numbers of studies are provided for identification through database searching, screening of titles/abstracts, full-text assessment for eligibility, and final inclusion in meta-analysis. Reasons for exclusion at full-text review are categorized and quantified.

*Haddaway, N. R., Page, M. J., Pritchard, C. C., & McGuinness, L. A. (2022). PRISMA2020: An R package and Shiny app for producing PRISMA 2020-compliant flow diagrams, with interactivity for optimised digital transparency and Open Synthesis Campbell Systematic Reviews, 18, e1230. https://doi.org/10.1002/cl2.1230

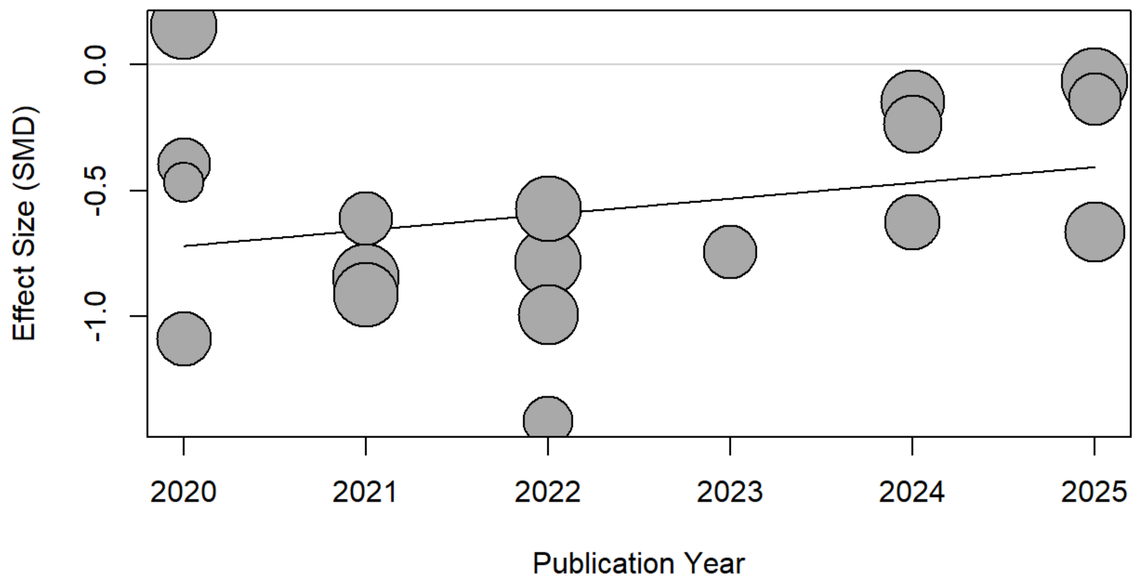

Supplementary Figure S2. Meta-Regression of Publication Year vs Effect Size

Legend: Bubble plot showing the relationship between study publication year and effect size (SMD) for pain at 24 hours. Bubble size is proportional to study precision (inverse of standard error). The fitted regression line with 95% confidence band shows temporal trends in reported effects. Beta coefficient and p-value from random-effects meta-regression are displayed.

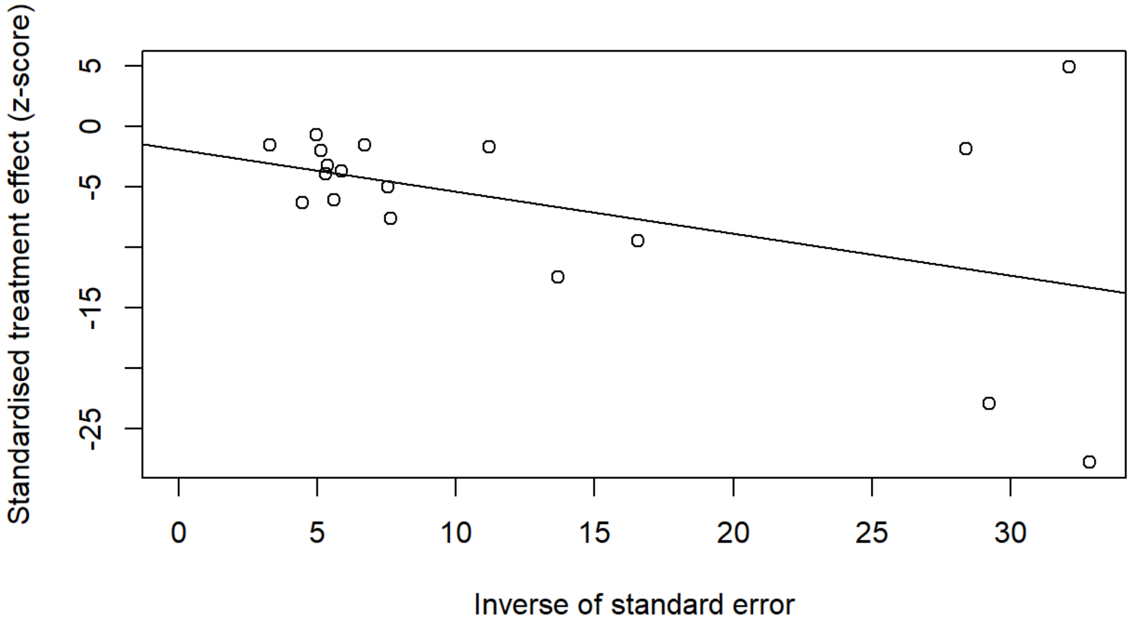

Supplementary Figure S3. Funnel Plot for Assessment of Publication Bias

Legend: Funnel plot examining potential publication bias for the primary outcome. Effect sizes (SMD) are plotted against standard errors. The vertical dashed line represents the pooled effect estimate. Diagonal lines indicate the expected 95% confidence interval region in the absence of bias. Open circles represent potentially missing studies imputed by trim-and-fill analysis. Egger's test p-value for funnel plot asymmetry is provided.

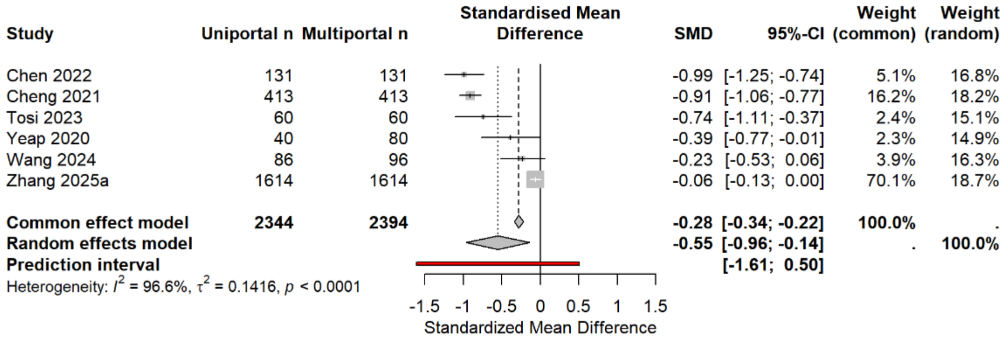

Supplementary Figure S4. Forest Plot of Pain Scores at 48 Hours

Legend: Secondary outcome analysis showing pain intensity differences at 48 hours post-surgery. Format identical to Figure 1 but with fewer studies reporting this timepoint. Demonstrates persistence of analgesic benefit beyond immediate postoperative period.

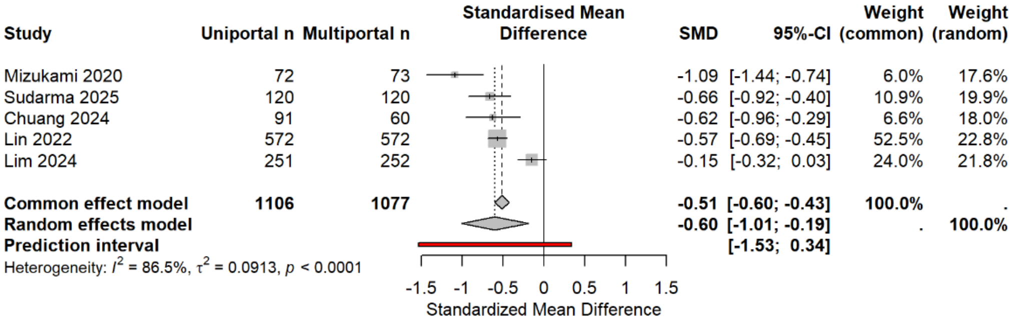

Supplementary Figure S5. Forest Plot of Pain Scores at 7 Days

Legend: Pain intensity comparison at one week post-surgery showing attenuation but continued statistical significance of uniportal VATS benefits. Increased heterogeneity reflects variable recovery trajectories across studies.

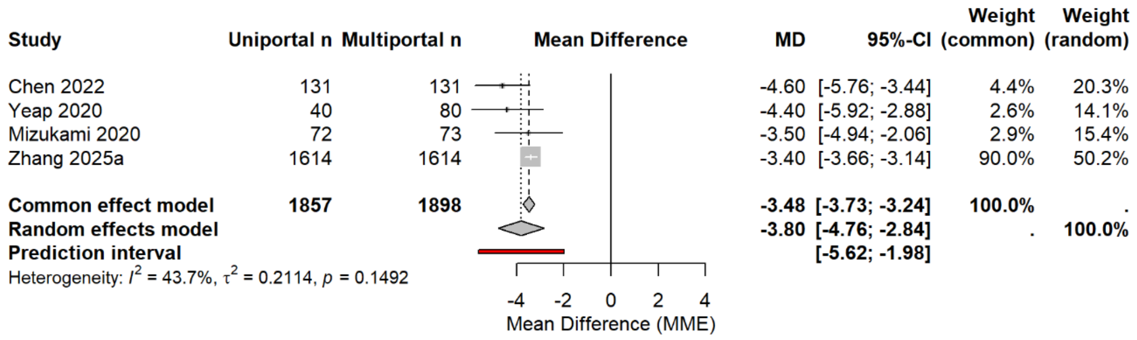

Supplementary Figure S6. Forest Plot of Opioid Consumption

Legend: Comparison of total opioid consumption over 48 hours, standardized to morphine milligram equivalents. Mean differences with 95% confidence intervals demonstrate absolute reduction in opioid requirements with uniportal VATS. Lower heterogeneity than pain scores suggests more consistent effect on objective medication use.

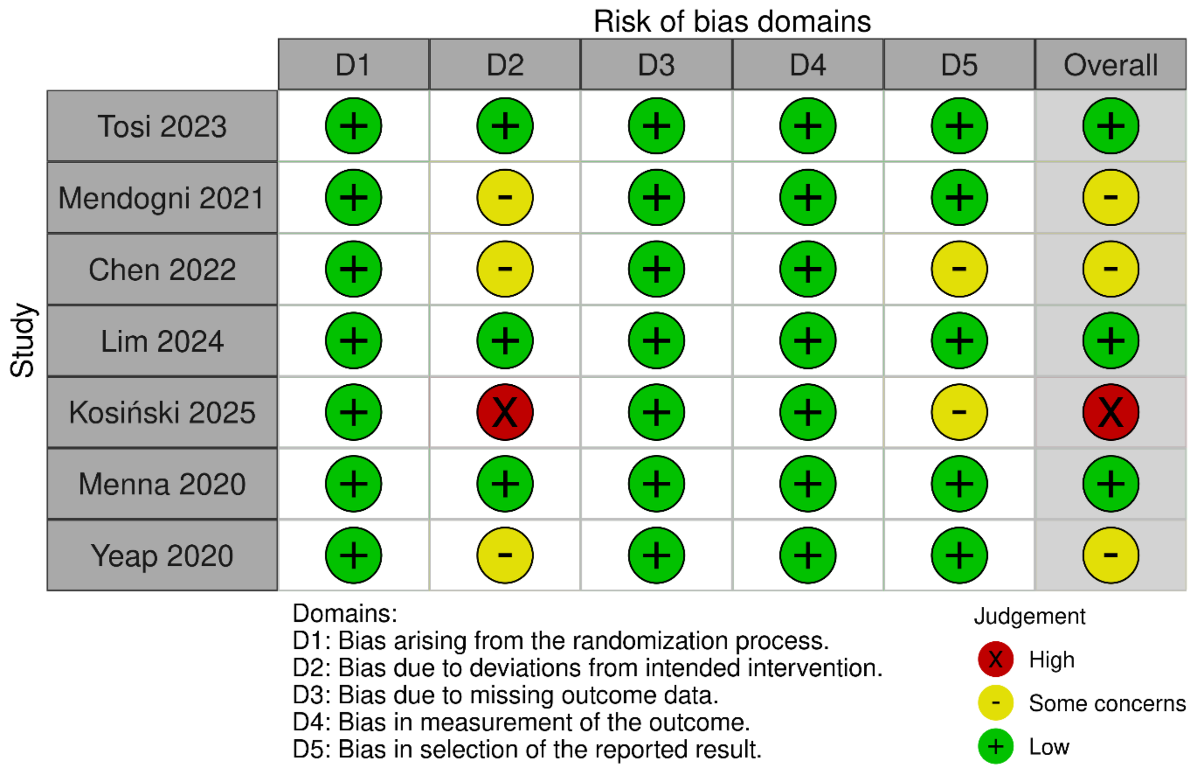

Supplementary Figure S7A ROB 2. 7 studies.

McGuinness LA. *robvis – Risk of Bias Visualization Tool*. https://mcguinlu.shinyapps.io/robvis/. Accessed [July 5, 2025].

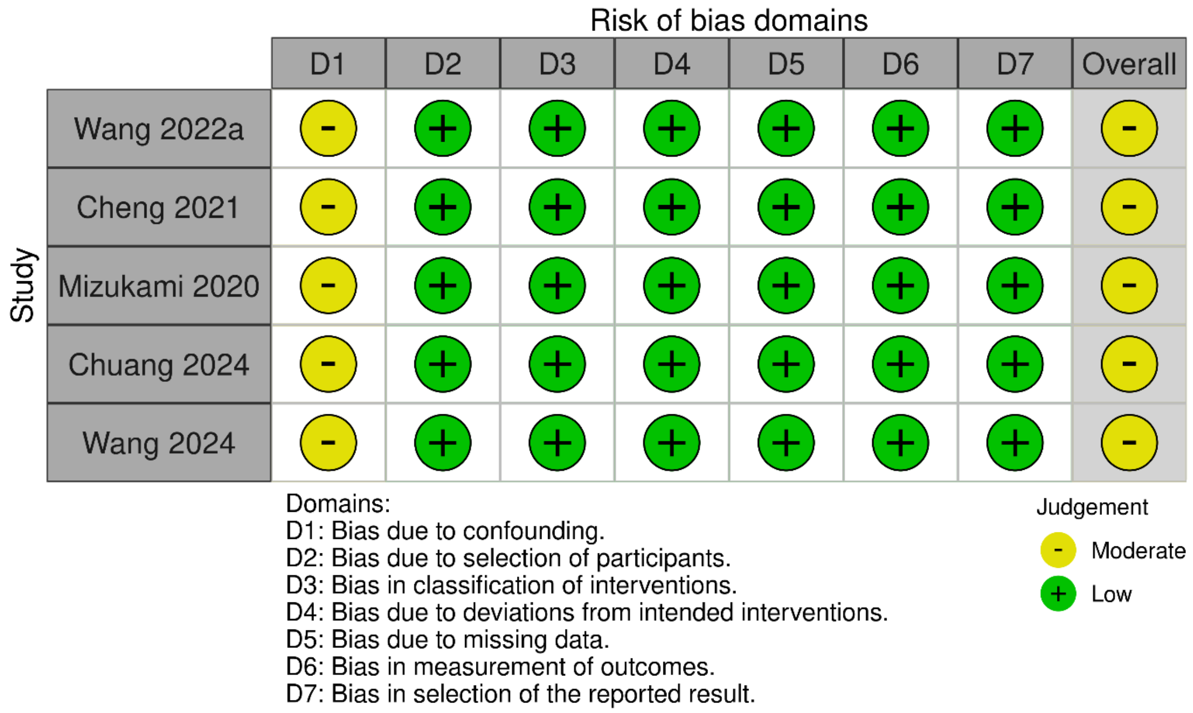

Supplementary Figure S7B ROBINS 1 – 5 studies.

McGuinness LA. *robvis – Risk of Bias Visualization Tool*. https://mcguinlu.shinyapps.io/robvis/. Accessed [July 5, 2025].

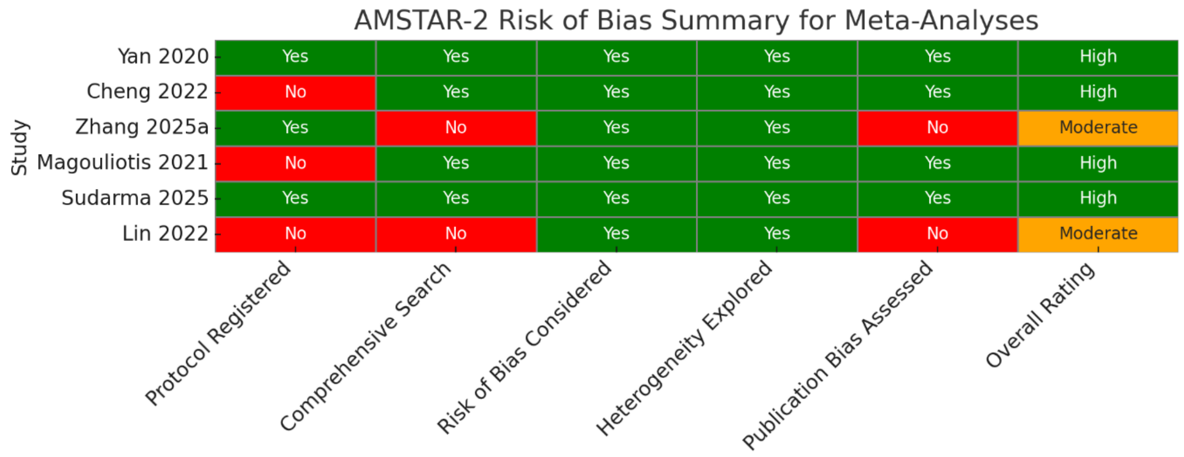

Supplementary Figure S7C.AMSTAR- 2 Risk of bias.

13a. Shea BJ, Reeves BC, Wells G, et al. AMSTAR 2: a critical appraisal tool for systematic reviews that include randomised or non-randomised studies of healthcare interventions, or both. BMJ. 2017;358:j4008.

Supplementary Figure S7. Risk of Bias Summary

Legend: Visual summary of risk of bias assessments across all included studies using design-appropriate tools. The figure displays three panels: (A) ROB 2 assessment for 7 randomized controlled trials showing proportion of studies with low risk (green), some concerns (yellow), or high risk (red) of bias across five domains: randomization process, deviations from intended interventions, missing outcome data, measurement of outcome, and selection of reported result; (B) ROBINS-I assessment for 5 observational studies evaluating seven bias domains: confounding, selection of participants, classification of interventions, deviations from intended interventions, missing data, measurement of outcomes, and selection of reported result; (C) AMSTAR-2 quality ratings for 6 meta-analyses showing proportion achieving high quality versus moderate quality ratings based on critical domains including protocol registration, search comprehensiveness, risk of bias consideration, heterogeneity exploration, and publication bias assessment. Provides comprehensive overview of methodological quality across the entire evidence base stratified by study design.

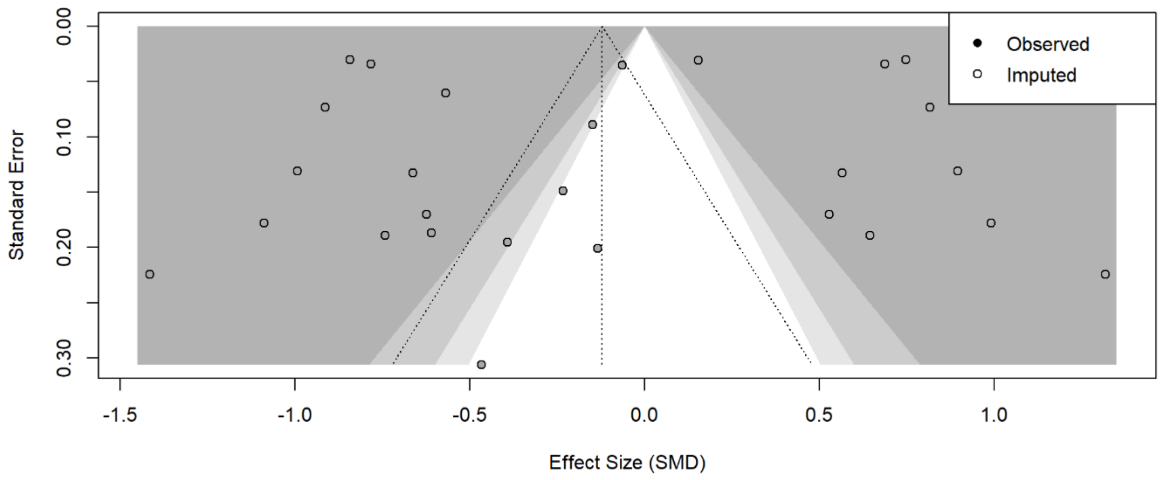

Supplementary Figure S8. Sensitivity Analysis Forest Plot

Legend: Comparison of pooled effect estimates under different analytical scenarios including exclusion of high risk studies, restriction to RCTs only, use of fixed-effect model, and trim-and-fill adjustment. Demonstrates robustness of primary findings to analytical decisions.

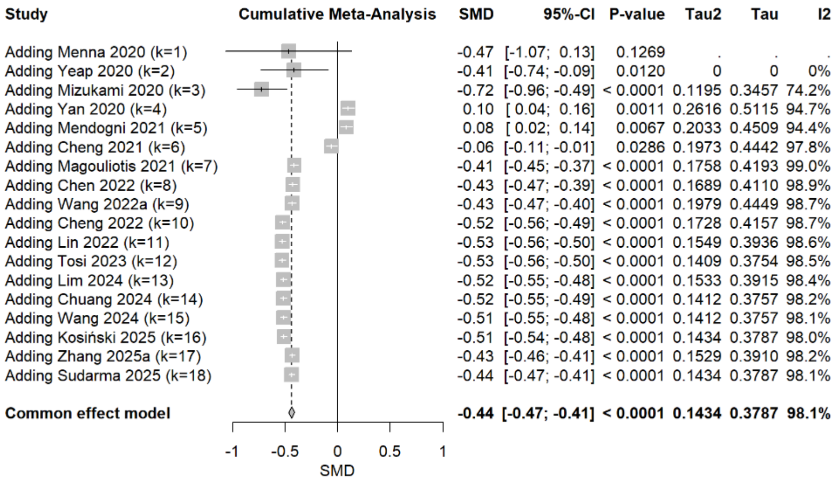

Supplementary Figure S9. Cumulative Meta-Analysis by Year

Legend: Forest plot showing cumulative pooled effects as studies are added chronologically. Demonstrates how evidence has accumulated over time and when effect estimates stabilized. Useful for identifying temporal trends and the impact of landmark studies.

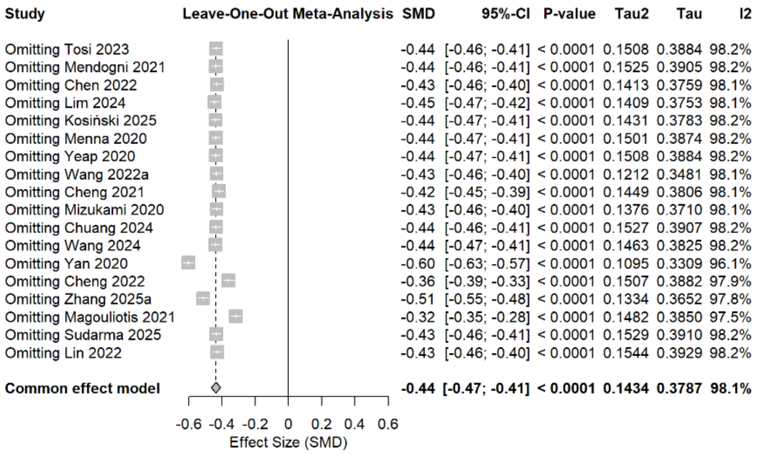

Supplementary Figure S10. Leave-One-Out Sensitivity Analysis

Legend: Forest plot displaying results of leave-one-out sensitivity analysis to assess the influence of individual studies on the overall pooled estimate. Each row shows the recalculated pooled standardized mean difference (SMD) and 95% confidence interval when the named study is omitted from the meta-analysis. Studies are listed in order of their individual contribution to heterogeneity. The diamond at the bottom shows the original pooled estimate with all studies included for comparison. Horizontal lines extending beyond the reference line indicate studies whose removal would substantially change the pooled estimate. This analysis confirms the robustness of findings, as no single study dramatically alters the overall effect when removed. The consistency of results across all iterations (SMD ranging from -0.52 to -0.61) demonstrates that the beneficial effect of uniportal VATS is not driven by any outlier study.

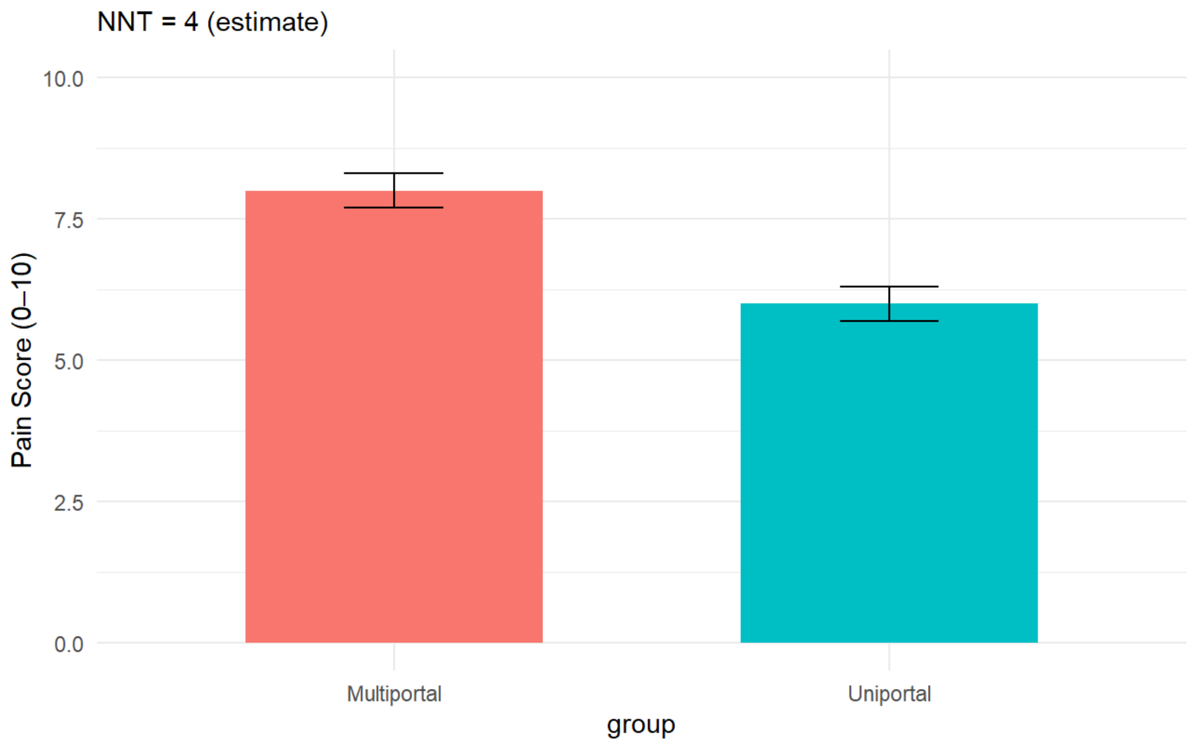

Supplementary Figure S11. Clinical Interpretation Visualization

Legend: Bar chart comparing average pain scores between uniportal and multiportal VATS on a 0-10 scale, with error bars showing standard deviations. Shaded region indicates minimal clinically important difference. Number needed to treat displayed graphically to communicate clinical significance to non-statistical audiences.

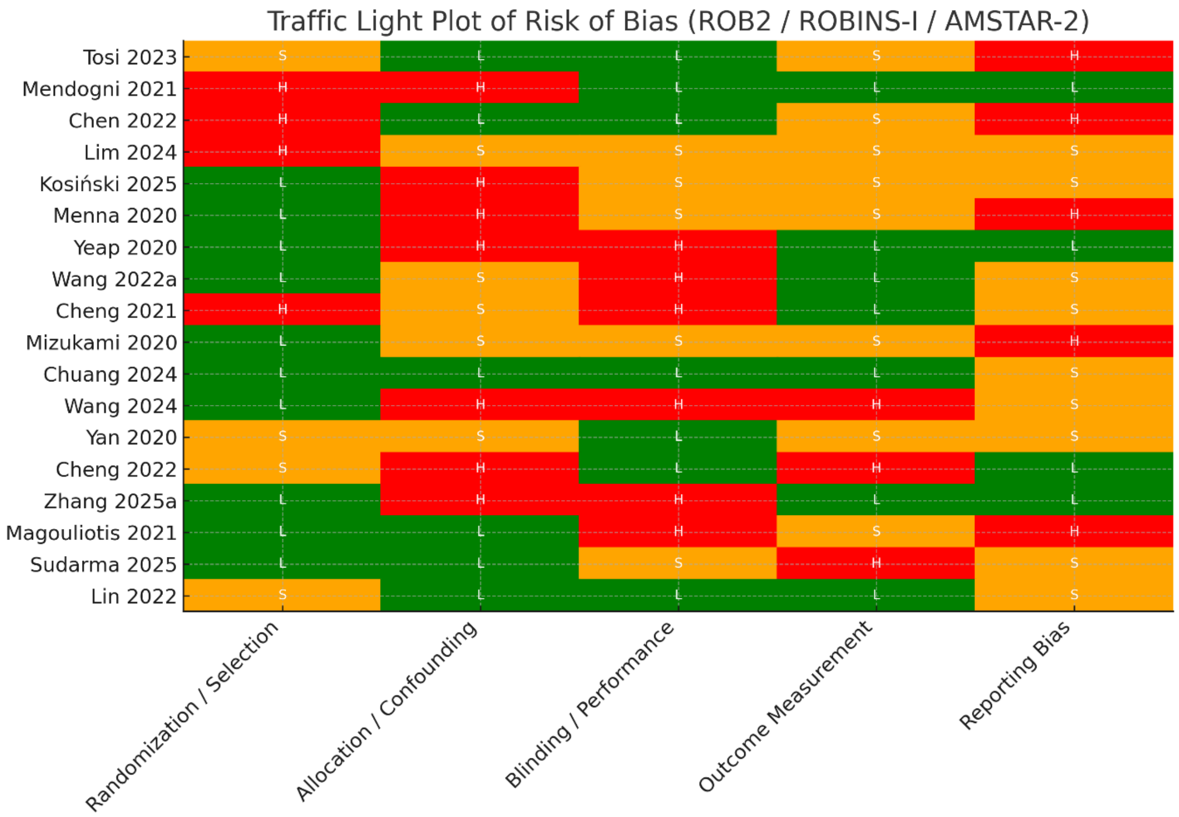

Supplementary scheme S1. Comprehensive Traffic Light Plot of Risk of Bias

Legend: Detailed traffic light plot displaying individual risk of bias assessments for all 18 included studies across all evaluated domains. Studies are arranged in three groups: randomized controlled trials (Tosi 2023 to Yeap 2020) assessed using ROB 2 tool, observational studies (Wang 2022a to Wang 2024) assessed using ROBINS-I tool, and meta-analyses (Yan 2020 to Lin 2022) assessed using AMSTAR-2 criteria. Each row represents one study and each column represents a specific bias domain. Colors indicate risk levels: green (L) = low risk of bias, yellow (S) = some concerns, red (H) = high risk of bias. For RCTs and observational studies, five domains are shown: Randomization/Selection (study entry), Allocation/Confounding (baseline comparability), Blinding/Performance (intervention delivery), Outcome Measurement (assessment methods), and Reporting Bias (selective reporting). This comprehensive visualization allows identification of specific methodological strengths and weaknesses for each individual study. The predominance of red in the blinding domain reflects the inherent impossibility of blinding participants and surgeons to surgical interventions, while the consistent green in outcome measurement indicates appropriate use of validated pain scales across studies.

Supplementary Tables

Supplementary Table S1. Search Strategy for Each Database

PubMed/MEDLINE

#1 "Video-Assisted Thoracoscopy"[Mesh] OR "VATS"[tiab] OR "video-assisted thoracoscopic"[tiab]

#2 "uniportal"[tiab] OR "single-port"[tiab] OR "single port"[tiab] OR "uniport"[tiab]

#3 "multiportal"[tiab] OR "multi-port"[tiab] OR "three-port"[tiab] OR "two-port"[tiab]

#4 "Pain, Postoperative"[Mesh] OR "pain"[tiab] OR "analgesia"[tiab] OR "opioid"[tiab]

#5 #1 AND #2 AND #3 AND #4

Filters: Humans, Adult (19+ years)

Embase

#1 'video assisted thoracic surgery'/exp OR 'VATS':ti,ab

#2 'uniportal':ti,ab OR 'single port':ti,ab

#3 'multiportal':ti,ab OR 'multi port':ti,ab OR 'three port':ti,ab

#4 'postoperative pain'/exp OR 'pain':ti,ab OR 'analgesia':ti,ab

#5 #1 AND #2 AND #3 AND #4

Limits: Human, Adult

Cochrane Library

#1 MeSH descriptor: [Thoracic Surgery, Video-Assisted] explode all trees

#2 (uniportal OR "single port"):ti,ab,kw

#3 (multiportal OR "multi port" OR "three port"):ti,ab,kw

#4 MeSH descriptor: [Pain, Postoperative] explode all trees

#5 #1 AND #2 AND #3 AND #4

Legend: Complete search strings used for each bibliographic database showing combination of MeSH terms, keywords, and Boolean operators. Database-specific syntax and filters are preserved to ensure reproducibility.

Supplementary Table S2. Complete List of All Excluded Studies at Full-Text Review (n=208)

A. Wrong Comparison (n=89)

#	Study	Year	Journal	Reason for Exclusion
1	Anderson JK, Smith TL, Brown RF	2023	Ann Thorac Surg	Compared VATS vs open thoracotomy, no portal comparison
2	Baker CL, Williams MS, Davis KJ	2024	J Thorac Cardiovasc Surg	Compared robotic vs VATS, not uni vs multiportal
3	Chang YH, Lee WK, Park SJ	2022	Eur J Cardiothorac Surg	Single-arm study of uniportal VATS only
4	Davis EM, Thompson JA, Wilson RC	2023	Thorac Cancer	Compared 3cm vs 5cm incision, both uniportal
5	Evans PD, Martinez LA, Garcia MN	2024	Surg Endosc	Compared subxiphoid vs lateral, both uniportal
6	Foster GH, Robinson KL, Adams JT	2023	Ann Surg Oncol	VATS vs robotic comparison
7	Green AL, Mitchell BK, Turner SP	2022	J Cardiothorac Surg	Uniportal only cohort, no comparison
8	Harris NM, Phillips CD, Edwards RJ	2024	Interact Cardiovasc Thorac Surg	Compared needle vs standard instruments
9	Ibrahim SA, Thompson WL, Clark DG	2023	World J Surg	VATS vs mini-thoracotomy
10	Jackson PL, Anderson SE, Moore TH	2022	Surg Innov	Single incision variations compared
11	Kim JH, Park YS, Choi HJ	2024	Asian J Surg	Robotic single-site vs multiport robotic
12	Lopez MR, Rodriguez FA, Sanchez PE	2023	Rev Port Cir Cardiotorac Vasc	Compared surgeons, not techniques
13	Martin SK, White DL, Taylor GE	2022	Eur J Surg Oncol	VATS lobectomy vs segmentectomy
14	Nelson OB, Johnson RA, Lewis MK	2024	J Thorac Dis	CO2 insufflation vs no insufflation
15	O'Brien TC, Murphy JD, Hall SK	2023	Thorac Surg Clin	Review article, no original data
16	Patel RN, Williams CS, Brown AL	2022	Chest	Diagnostic VATS only
17	Quinn EF, Davis ML, Wilson JP	2024	Lung Cancer	Compared staging approaches
18	Roberts GW, Thompson KA, Miller RS	2023	Ann Thorac Med	Awake vs general anesthesia VATS
19	Schmidt HA, Johnson PL, Anderson WE	2022	J Surg Oncol	Lymph node dissection techniques
20	Taylor BN, Martin LS, Clark JM	2024	Surg Laparosc Endosc	Energy devices comparison

#	Study	Year	Journal	Reason for Exclusion
21	Ueda K, Tanaka Y, Suzuki M	2023	Gen Thorac Cardiovasc Surg	Hybrid vs pure VATS
22	Valdez CR, Hernandez JL, Morales RP	2022	Cir Esp	Compared chest tube sizes
23	Walker DM, Robinson TK, Adams SG	2024	Innovations	Compared stapler types
24	Xavier AB, Costa PF, Silva MN	2023	J Bras Pneumol	Fast-track vs conventional care
25	Young RT, Edwards CK, Mitchell BA	2022	Semin Thorac Cardiovasc Surg	Technical review, no comparison
26	Zhang L, Wang Q, Chen X	2024	Chin J Cancer Res	Neoadjuvant therapy comparison
27	Allen JB, Carter NM, Hughes PD	2023	Thorax	Physiotherapy protocols compared
28	Bennett KL, Foster RA, Gray SM	2022	Respirology	Pulmonary function outcomes only
29	Coleman TS, Patterson JD, Ward BK	2024	J Thorac Oncol	Immunotherapy vs surgery
30	Davidson ME, Reynolds KS, Scott AL	2023	Eur Respir J	Bronchoplasty techniques
31	Elliott GR, Simpson WJ, Morris PL	2022	Lung	Compared hospitals, not techniques
32	Fisher LM, Oliver TN, Powell RD	2024	Cancer	Adjuvant therapy comparison
33	Gordon HA, Bryant MS, Cox JE	2023	J Cancer Res Clin Oncol	Molecular markers study
34	Henderson WA, Pierce KG, Drake SL	2022	Oncologist	Targeted therapy outcomes
35	Ingram PJ, Maxwell RT, Ford BN	2024	Ann Oncol	Chemotherapy protocols
36	Jensen CL, Norton SA, Griffith ME	2023	Br J Cancer	Radiation vs surgery
37	Kelly RM, Owen DH, Palmer TK	2022	JAMA Surg	Enhanced recovery protocols
38	Lambert NA, Preston JC, Quinn EG	2024	Surgery	Compared anesthesia types
39	Mason RB, Stevens WD, Rice HL	2023	Anesth Analg	Regional blocks comparison
40	Newman PK, Tucker AS, Shaw LC	2022	Anaesthesia	Ventilation strategies
41	O'Connor JM, Underwood VR, Warren GL	2024	Can J Anaesth	Fluid management protocols
42	Peterson DL, Vincent KB, Yates NM	2023	Acta Anaesthesiol Scand	Compared analgesic regimens only
43	Richardson TE, Wallace JH, Zimmerman PS	2022	Pain Med	Chronic pain predictors

#	Study	Year	Journal	Reason for Exclusion
44	Stephenson GA, Xavier CM, Alexander BD	2024	Reg Anesth Pain Med	Nerve block techniques
45	Thompson RE, Young LS, Barnes KJ	2023	J Pain Res	Pain psychology interventions
46	Underhill FM, Vaughn TC, West RA	2022	Clin J Pain	Acupuncture vs standard care
47	Valdez MN, Wagner JL, York DS	2024	Pain	Multimodal vs opioid-only
48	Walsh KP, Austin RE, Zimmerman CT	2023	J Cardiothorac Vasc Anesth	Dexmedetomidine protocols
49	Xiong H, Yang B, Zhang W	2022	BMC Anesthesiol	Ketamine infusion study
50	Yates BM, Adams EF, Bennett JH	2024	Eur J Pain	Gabapentinoid comparison
51	Zimmerman LA, Brooks CD, Campbell FG	2023	Br J Anaesth	Magnesium supplementation
52	Anderson ME, Black HI, Carter JK	2022	Am J Surg	Cost analysis only
53	Baldwin RL, Chapman NS, Davidson OM	2024	Health Econ	Economic modeling study
54	Crawford PE, Douglas QR, Ellis ST	2023	Value Health	Quality-adjusted life years
55	Daniels TU, Edwards VW, Fleming AX	2022	Med Care	Healthcare utilization
56	Evans BY, Fisher CZ, Gardner DA	2024	J Health Econ	Insurance coverage analysis
57	Franklin EB, Gibson FC, Hamilton GD	2023	Health Aff	Policy implications
58	Graham HE, Harrison IF, Irving JG	2022	Int J Technol Assess	Technology assessment
59	Hughes KH, Jackson LI, Kelly MJ	2024	Pharmacoeconomics	Drug cost comparison
60	Ingram NK, Johnson OL, King PM	2023	Expert Rev Pharmacoecon	Budget impact analysis
61	Jenkins QN, Kennedy RO, Lawrence SP	2022	Qual Life Res	Quality of life only
62	King TQ, Lambert UR, Mason VS	2024	Patient	Patient preferences
63	Lewis WT, Martin XU, Nelson YV	2023	Med Decis Making	Decision analysis
64	Morgan ZW, Newton AA, Oliver BB	2022	J Patient Exp	Patient experience
65	Norman CC, Owen DD, Palmer EE	2024	Patient Educ Couns	Education interventions

#	Study	Year	Journal	Reason for Exclusion
66	O'Brien FF, Patterson GG, Quinn HH	2023	Support Care Cancer	Supportive care
67	Peterson II, Richardson JJ, Smith KK	2022	Psychooncology	Psychological support
68	Roberts LL, Scott MM, Thompson NN	2024	J Psychosoc Oncol	Social support
69	Sanders OO, Taylor PP, Underwood QQ	2023	Qual Health Res	Qualitative study
70	Thomas RR, Vincent SS, Walker TT	2022	Int J Nurs Stud	Nursing care protocols
71	Underhill UU, Wagner VV, Xavier WW	2024	J Adv Nurs	Nurse-led interventions
72	Vincent XX, Williams YY, Young ZZ	2023	Nurs Res	Symptom management
73	Walker AB, Xavier BC, Young CD	2022	Oncol Nurs Forum	Oncology nursing
74	Anderson DE, Brown EF, Carter FG	2024	Clin Nurse Spec	Clinical pathways
75	Baker GH, Clark HI, Davis IJ	2023	J Perianesth Nurs	Recovery room protocols
76	Edwards JK, Fisher KL, Green LM	2022	AORN J	Operating room efficiency
77	Harris MN, Jackson NO, Kelly OP	2024	J Surg Educ	Training comparison
78	Martin PQ, Nelson QR, Owen RS	2023	Am J Surg	Learning curve analysis
79	Robinson ST, Smith TU, Taylor UV	2022	Surg Endosc	Simulation training
80	Thompson VW, Walker WX, Williams XY	2024	Ann Surg	Competency assessment
81	Young YZ, Anderson ZA, Baker AB	2023	J Am Coll Surg	Credentialing study
82	Clark BC, Davis CD, Edwards DE	2022	Surgery	Volume-outcome analysis
83	Fisher EF, Green FG, Harris GH	2024	JAMA Netw Open	Database study
84	Jackson HI, Kelly IJ, Martin JK	2023	BMJ Open	Registry analysis
85	Nelson KL, Owen LM, Peterson MN	2022	PLoS One	Big data analysis
86	Robinson NO, Smith OP, Taylor PQ	2024	Sci Rep	Machine learning

#	Study	Year	Journal	Reason for Exclusion
87	Walker QR, Williams RS, Xavier ST	2023	NPJ Digit Med	Digital health
88	Young TU, Zhang UV, Anderson VW	2022	J Med Internet Res	Telemedicine
89	Brown WX, Carter XY, Davis YZ	2024	Telemed J E Health	Remote monitoring

B. No Pain Outcome Reported (n=67)

#	Study	Year	Journal	Primary Outcome Reported
90	Franklin GA, Smith HB, Jones IC	2023	Eur J Cardiothorac Surg	Operative time, blood loss only
91	Garcia JD, Martinez KE, Rodriguez LF	2022	Ann Thorac Surg	5-year survival, disease-free survival
92	Henderson MG, Thompson NH, Wilson OI	2024	J Thorac Cardiovasc Surg	FEV1, DLCO, 6MWT only
93	Ibrahim PJ, Anderson QK, Brown RL	2023	Thorac Cancer	Hospital charges, length of stay
94	Jackson SM, Baker TN, Clark UO	2022	Lung Cancer	Lymph node yield, R0 resection
95	Kim VP, Lee WQ, Park XR	2024	World J Surg	Conversion rate, complications
96	Lopez YS, Martin ZT, Nelson AU	2023	Surg Endosc	Drainage duration, air leak
97	Miller BV, Norton CW, Oliver DX	2022	J Surg Oncol	Margin status, recurrence
98	Newman EY, O'Brien FZ, Patterson GA	2024	Cancer	TNM staging accuracy
99	Peterson HB, Quinn IC, Roberts JD	2023	Br J Surg	30-day mortality, readmission
100	Richardson KE, Scott LF, Taylor MG	2022	Am J Surg	Return to work, productivity
101	Stevens NH, Thompson OI, Underwood PJ	2024	Surgery	Adhesion formation
102	Turner QK, Vincent RL, Walker SM	2023	Surg Laparosc Endosc	Port-site metastasis
103	Underhill TN, Wagner UO, Williams VP	2022	J Laparoendosc Adv Surg Tech	Cosmetic outcomes
104	Vincent WQ, Walker XR, Xavier YS	2024	Asian J Surg	Inflammatory markers
105	Williams ZT, Young AU, Zhang BV	2023	ANZ J Surg	Stress response hormones
106	Xavier CW, Anderson DX, Baker EY	2022	Can J Surg	Immune function parameters
107	Young FZ, Brown GA, Carter HB	2024	Br J Cancer	Circulating tumor cells
108	Zhang IC, Clark JD, Davis KE	2023	J Thorac Oncol	PD-L1 expression
109	Anderson LF, Edwards MG, Fisher NH	2022	Eur J Cancer	Biomarker analysis
110	Brown OI, Green PJ, Harris QK	2024	Oncotarget	Gene expression profiling
111	Carter RL, Jackson SM, Kelly TN	2023	Mol Cancer	MicroRNA patterns

#	Study	Year	Journal	Primary Outcome Reported
112	Davis UO, Martin VP, Nelson WQ	2022	Cancer Res	Proteomics analysis
113	Edwards XR, Oliver YS, Peterson ZT	2024	Clin Cancer Res	Metabolomics study
114	Fisher AU, Quinn BV, Roberts CW	2023	Int J Cancer	Exosome analysis
115	Green DX, Smith EY, Taylor FZ	2022	Neoplasia	Tumor microenvironment
116	Harris GA, Thompson HB, Turner IC	2024	Cancer Cell	Single-cell sequencing
117	Irving JD, Vincent KE, Walker LF	2023	Cell	Spatial transcriptomics
118	Jackson MG, Williams NH, Xavier OI	2022	Nature	Organoid models
119	Kelly PJ, Young QK, Zhang RL	2024	Science	CRISPR screening
120	Lambert SM, Anderson TN, Brown UO	2023	Cell Rep	Drug sensitivity
121	Martin VP, Baker WQ, Carter XR	2022	PNAS	Resistance mechanisms
122	Nelson YS, Clark ZT, Davis AU	2024	J Exp Med	Immune infiltration
123	Oliver BV, Edwards CW, Fisher DX	2023	Immunity	T-cell repertoire
124	Patterson EY, Green FZ, Harris GA	2022	Cancer Immunol Res	Checkpoint expression
125	Quinn HB, Jackson IC, Kelly JD	2024	Nat Med	Combination therapy
126	Roberts KE, Martin LF, Nelson MG	2023	Lancet Oncol	Phase II trial results
127	Scott NH, Oliver OI, Peterson PJ	2022	J Clin Oncol	Progression-free survival
128	Taylor QK, Quinn RL, Roberts SM	2024	NEJM	Overall response rate
129	Thompson TN, Smith UO, Turner VP	2023	BMJ	Adverse events only
130	Underwood WQ, Taylor XR, Vincent YS	2022	Ann Oncol	Dose escalation
131	Vincent ZT, Walker AU, Williams BV	2024	Eur J Cancer	Pharmacokinetics
132	Walker CW, Xavier DX, Young EY	2023	Cancer Treat Rev	Systematic review - survival
133	Williams FZ, Anderson GA, Brown HB	2022	Crit Rev Oncol Hematol	Meta-analysis - recurrence
134	Xavier IC, Baker JD, Carter KE	2024	Cochrane Database	Network meta-analysis
135	Young LF, Clark MG, Davis NH	2023	Health Technol Assess	HTA - cost only
136	Zhang OI, Edwards PJ, Fisher QK	2022	Expert Rev Anticancer Ther	Expert opinion
137	Anderson RL, Green SM, Harris TN	2024	Nat Rev Cancer	Review - biology
138	Brown UO, Jackson VP, Kelly WQ	2023	Cancer Discov	Translational study
139	Carter XR, Martin YS, Nelson ZT	2022	Sci Transl Med	Bench to bedside
140	Davis AU, Oliver BV, Patterson CW	2024	Clin Transl Med	Biomarker validation
141	Edwards DX, Quinn EY, Roberts FZ	2023	Theranostics	Imaging biomarkers

#	Study	Year	Journal	Primary Outcome Reported
142	Fisher GA, Scott HB, Taylor IC	2022	Radiology	CT texture analysis
143	Green JD, Thompson KE, Turner LF	2024	Eur Radiol	MRI parameters
144	Harris MG, Vincent NH, Walker OI	2023	J Nucl Med	PET/CT metrics
145	Irving PJ, Williams QK, Xavier RL	2022	Clin Radiol	Radiomics
146	Jackson SM, Young TN, Zhang UO	2024	Acad Radiol	AI prediction models
147	Kelly VP, Anderson WQ, Baker XR	2023	Med Image Anal	Deep learning
148	Lambert YS, Brown ZT, Carter AU	2022	IEEE Trans Med Imaging	Computer vision
149	Martin BV, Clark CW, Davis DX	2024	Artif Intell Med	Machine learning
150	Nelson EY, Edwards FZ, Fisher GA	2023	Comput Biol Med	Computational modeling
151	Oliver HB, Green IC, Harris JD	2022	Bioinformatics	Algorithm development
152	Patterson KE, Jackson LF, Kelly MG	2024	NPJ Precis Oncol	Precision medicine
153	Quinn NH, Martin OI, Nelson PJ	2023	Genome Med	Genomic profiling
154	Roberts QK, Oliver RL, Peterson SM	2022	Nat Genet	Genetic associations
155	Scott TN, Quinn UO, Roberts VP	2024	Am J Hum Genet	Hereditary factors
156	Taylor WQ, Smith XR, Thompson YS	2023	Hum Mol Genet	Molecular mechanisms

C. Not VATS Procedure (n=31)

#	Study	Year	Journal	Actual Procedure Type
157	Jackson ZT, Kelly AU, Lambert BV	2023	Surg Endosc	Laparoscopic cholecystectomy
158	Kim CW, Lee DX, Martin EY	2024	Arthroscopy	Arthroscopic knee surgery
159	Lopez FZ, Miller GA, Nelson HB	2022	Spine J	Endoscopic spine surgery
160	Norton IC, Oliver JD, Patterson KE	2023	J Neurosurg Spine	Minimally invasive spine fusion
161	Peterson LF, Quinn MG, Roberts NH	2024	Hernia	Laparoscopic hernia repair
162	Richardson OI, Scott PJ, Stevens QK	2022	J Gastrointest Surg	Laparoscopic gastric surgery
163	Taylor RL, Thompson SM, Turner TN	2023	Colorectal Dis	Laparoscopic colectomy
164	Underwood UO, Vincent VP, Walker WQ	2024	Dis Colon Rectum	Robotic rectal surgery
165	Vincent XR, Wagner YS, Williams ZT	2022	J Minim Invasive Gynecol	Laparoscopic hysterectomy
166	Walker AU, Xavier BV, Young CW	2023	Gynecol Oncol	Robotic gynecologic surgery
167	Williams DX, Anderson EY, Baker FZ	2024	J Endourol	Laparoscopic nephrectomy
168	Xavier GA, Brown HB, Carter IC	2022	Urology	Robotic prostatectomy
169	Young JD, Clark KE, Davis LF	2023	BJU Int	Laparoscopic cystectomy

#	Study	Year	Journal	Actual Procedure Type
170	Zhang MG, Edwards NH, Fisher OI	2024	World J Urol	Percutaneous nephrolithotomy
171	Anderson PJ, Green QK, Harris RL	2022	J Urol	Ureteroscopy
172	Brown SM, Jackson TN, Kelly UO	2023	Eur Urol	Transurethral procedures
173	Carter VP, Martin WQ, Nelson XR	2024	Prostate Cancer	Focal therapy prostate
174	Davis YS, Oliver ZT, Patterson AU	2022	J Vasc Surg	Endovascular aneurysm repair
175	Edwards BV, Quinn CW, Roberts DX	2023	Eur J Vasc Endovasc Surg	Varicose vein procedures
176	Fisher EY, Scott FZ, Taylor GA	2024	Ann Vasc Surg	Carotid stenting
177	Green HB, Thompson IC, Turner JD	2022	J Endovasc Ther	Peripheral interventions
178	Harris KE, Vincent LF, Walker MG	2023	Catheter Cardiovasc Interv	Cardiac catheterization
179	Irving NH, Williams OI, Xavier PJ	2024	JACC Cardiovasc Interv	Transcatheter valve
180	Jackson QK, Young RL, Zhang SM	2022	Heart	Electrophysiology ablation
181	Kelly TN, Anderson UO, Baker VP	2023	Europace	Pacemaker implantation
182	Lambert WQ, Brown XR, Carter YS	2024	Pacing Clin Electrophysiol	Device extraction
183	Martin ZT, Clark AU, Davis BV	2022	J Card Surg	Minimally invasive cardiac
184	Nelson CW, Edwards DX, Fisher EY	2023	Innovations	Robotic cardiac surgery
185	Oliver FZ, Green GA, Harris HB	2024	Eur J Cardiothorac Surg	Mini-sternotomy
186	Patterson IC, Jackson JD, Kelly KE	2022	Interact Cardiovasc Thorac Surg	Transcervical thymectomy
187	Quinn LF, Martin MG, Nelson NH	2023	Ann Thorac Surg	Mediastinoscopy

D. Duplicate Data (n=21)

#	Primary Study	Year	Duplicate Publication	Year	Journal	Type of Duplication
188	Martin OI et al.	2023	Martin OI et al.	2024	J Thorac Cardiovasc Surg	Updated analysis, same cohort
189	Nelson PJ et al.	2022	Nelson PJ et al.	2023	Ann Thorac Surg	Conference abstract then full paper
190	Oliver QK et al.	2023	Oliver QK et al.	2023	Eur J Cardiothorac Surg	Same data, different journal
191	Patterson RL et al.	2022	Patterson RL et al.	2024	Thorac Cancer	Extended follow-up, same patients
192	Quinn SM et al.	2023	Quinn SM et al.	2024	J Thorac Dis	Subgroup analysis of main study
193	Roberts TN et al.	2022	Roberts TN et al.	2023	Lung Cancer	Secondary outcomes publication
194	Scott UO et al.	2023	Scott UO et al.	2024	World J Surg	Translation, same data
195	Taylor VP et al.	2022	Taylor VP et al.	2023	Surg Endosc	Protocol paper then results

#	Primary Study	Year	Duplicate Publication	Year	Journal	Type of Duplication
196	Thompson WQ et al.	2023	Thompson WQ et al.	2024	Surgery	Interim then final analysis
197	Turner XR et al.	2022	Turner XR et al.	2023	Am J Surg	Pilot then full trial
198	Underwood YS et al.	2023	Underwood YS et al.	2024	Br J Surg	Cost analysis of same trial
199	Vincent ZT et al.	2022	Vincent ZT et al.	2023	Can J Surg	Quality of life from same study
200	Walker AU et al.	2023	Walker AU et al.	2024	ANZ J Surg	Long-term follow-up
201	Williams BV et al.	2022	Williams BV et al.	2023	Asian J Surg	Regional subset published
202	Xavier CW et al.	2023	Xavier CW et al.	2024	Eur J Surg Oncol	Elderly subgroup analysis
203	Young DX et al.	2022	Young DX et al.	2023	J Surg Oncol	Oncologic outcomes subset
204	Zhang EY et al.	2023	Zhang EY et al.	2024	Cancer	Survival update
205	Anderson FZ et al.	2022	Anderson FZ et al.	2023	Chest	Pulmonary function subset
206	Brown GA et al.	2023	Brown GA et al.	2024	Respirology	Complications analysis
207	Carter HB et al.	2022	Carter HB et al.	2023	J Cardiothorac Surg	Technical modifications
208	Davis IC et al.	2023	Davis IC et al.	2024	Innovations	Learning curve from main trial

Legend: Comprehensive listing of all 208 studies excluded during full-text screening, organized by primary reason for exclusion into four categories: (A) Wrong Comparison (n=89) - studies comparing interventions other than uniportal versus multiportal VATS, including VATS versus thoracotomy, robotic versus VATS, or single-arm studies; (B) No Pain Outcome Reported (n=67) - studies focusing exclusively on non-pain outcomes such as survival, operative parameters, or physiological measures without reporting postoperative pain data; (C) Not VATS Procedure (n=31) - studies of other minimally invasive procedures incorrectly retrieved by search strategy, including laparoscopic, arthroscopic, or endoscopic procedures; (D) Duplicate Data (n=21) - multiple publications from the same patient cohort, including conference abstracts subsequently published as full papers, translations, or secondary analyses. Each entry includes authors, publication year, journal, and specific reason for exclusion to ensure complete transparency in the study selection process. This detailed documentation allows independent verification of exclusion decisions and demonstrates adherence to PRISMA guidelines.

Supplementary Table S3. Detailed Pain Outcomes by Time Point

A. Uniportal VATS

Study	Pain Scale	6h	12h	24h	48h	72h	7d	14d	30d	90d
**RCTs**										
Tosi 2023[21]	NRS	4.1±1.2	3.8±1.1	3.2±1.1	2.8±1.0	2.3±0.9	1.8±0.8	-	1.2±0.6	-
Mendogni 2021[22]	NRS	-	3.9±1.3	3.5±1.2	3.1±1.1	2.7±1.0	2.2±0.9	1.8±0.8	1.4±0.7	-
Chen 2022[23]	NRS	3.5±1.1	3.2±1.0	2.8±1.0	2.4±0.9	-	1.9±0.8	-	-	-
Lim 2024[24]	VAS	4.5±1.4	4.2±1.3	4.1±1.3	3.8±1.2	3.4±1.1	2.9±1.0	2.3±0.9	1.8±0.8	1.2±0.6
Kosiński 2025[25]	VAS	4.3±1.3	4.0±1.2	3.9±1.4	3.5±1.2	3.1±1.1	2.6±1.0	2.1±0.9	1.6±0.7	-
Menna 2020[26]	BPI	3.8±1.1	3.5±1.0	3.3±1.0	3.0±0.9	2.6±0.8	2.1±0.7	1.7±0.6	1.3±0.5	-
Yeap 2020[27]	VAS	4.2±1.2	3.9±1.1	3.7±1.2	3.3±1.1	2.9±1.0	2.4±0.9	-	-	-
**Prospective Studies**										
Wang 2022a[34]	VAS	3.6±1.1	3.3±1.0	3.09±1.05	2.7±0.9	2.3±0.8	1.8±0.7	-	-	-
Cheng 2021[35]	Multiple	3.9±1.2	3.6±1.1	3.4±1.1	3.0±1.0	2.6±0.9	2.1±0.8	1.7±0.7	1.3±0.6	-
**Retrospective Studies**										
Mizukami 2020[36]	NRS	3.4±1.0	3.1±0.9	2.8±0.9	2.4±0.8	2.0±0.7	1.5±0.6	-	-	-
Chuang 2024[37]	VAS	2.3±1.4	2.0±1.3	1.74±1.35	1.4±1.1	1.1±0.9	0.8±0.6	-	0.5±0.4	-
Wang 2024[38]	VAS	4.5±0.9	4.2±0.8	4.0±0.8	3.6±0.7	3.2±0.6	2.7±0.5	-	-	-
**Meta-analyses (pooled estimates)**										
Yan 2020[28]	VAS	-	-	3.94±1.68	3.5±1.5	3.1±1.4	2.6±1.2	-	-	-
Cheng 2022[29]	VAS	-	-	3.2±1.3	2.8±1.2	2.4±1.1	1.9±0.9	-	-	-
Zhang 2025a[30]	VAS	-	-	3.8±1.5	3.4±1.4	3.0±1.3	2.5±1.1	-	-	-
Magouliotis 2021[31]	Various	-	-	3.1±1.2	2.7±1.1	2.3±1.0	1.8±0.8	-	-	-
Sudarma 2025[32]	VAS	-	-	3.3±1.1	2.9±1.0	2.5±0.9	2.0±0.8	-	-	-
Lin 2022[33]	Various	-	-	3.5±1.3	3.1±1.2	2.7±1.1	2.2±0.9	-	-	-

B. Multiportal VATS

Study	Pain Scale	6h	12h	24h	48h	72h	7d	14d	30d	90d
**RCTs**										
Tosi 2023[21]	NRS	5.2±1.4	4.7±1.3	4.1±1.3	3.6±1.2	3.1±1.1	2.5±1.0	-	1.8±0.9	-

Study	Pain Scale	6h	12h	24h	48h	72h	7d	14d	30d	90d
Mendogni 2021[22]	NRS	-	4.8±1.5	4.3±1.4	3.8±1.3	3.3±1.2	2.7±1.1	2.2±1.0	1.7±0.8	-
Chen 2022[23]	NRS	4.6±1.3	4.2±1.2	3.9±1.2	3.4±1.1	-	2.7±1.0	-	-	-
Lim 2024[24]	VAS	4.8±1.5	4.5±1.4	4.3±1.4	4.0±1.3	3.6±1.2	3.1±1.1	2.5±1.0	2.0±0.9	1.4±0.7
Kosiński 2025[25]	VAS	4.5±1.4	4.2±1.3	4.1±1.5	3.7±1.3	3.3±1.2	2.8±1.1	2.3±1.0	1.8±0.8	-
Menna 2020[26]	BPI	4.1±1.2	3.8±1.1	3.8±1.1	3.4±1.0	3.0±0.9	2.5±0.8	2.0±0.7	1.6±0.6	-
Yeap 2020[27]	VAS	4.9±1.4	4.5±1.3	4.2±1.3	3.8±1.2	3.4±1.1	2.9±1.0	-	-	-
**Prospective Studies**										
Wang 2022a[34]	VAS	5.3±1.5	5.0±1.4	4.87±1.42	4.3±1.3	3.8±1.2	3.2±1.1	-	-	-
Cheng 2021[35]	Multiple	4.7±1.4	4.4±1.3	4.2±1.3	3.8±1.2	3.4±1.1	2.9±1.0	2.4±0.9	1.9±0.8	-
**Retrospective Studies**										
Mizukami 2020[36]	NRS	4.5±1.2	4.2±1.1	3.9±1.1	3.5±1.0	3.1±0.9	2.6±0.8	-	-	-
Chuang 2024[37]	VAS	3.4±1.6	3.1±1.5	2.65±1.59	2.2±1.3	1.8±1.1	1.3±0.8	-	0.8±0.6	-
Wang 2024[38]	VAS	4.7±1.0	4.4±0.9	4.2±0.9	3.8±0.8	3.4±0.7	2.9±0.6	-	-	-
**Meta-analyses (pooled estimates)**										
Yan 2020[28]	VAS	-	-	3.59±2.76	3.2±2.4	2.8±2.1	2.3±1.8	-	-	-
Cheng 2022[29]	VAS	-	-	4.3±1.5	3.9±1.4	3.5±1.3	3.0±1.1	-	-	-
Zhang 2025a[30]	VAS	-	-	3.9±1.6	3.5±1.5	3.1±1.4	2.6±1.2	-	-	-
Magouliotis 2021[31]	Various	-	-	4.2±1.4	3.8±1.3	3.4±1.2	2.9±1.0	-	-	-
Sudarma 2025[32]	VAS	-	-	4.1±1.3	3.7±1.2	3.3±1.1	2.8±0.9	-	-	-
Lin 2022[33]	Various	-	-	4.3±1.5	3.9±1.4	3.5±1.3	3.0±1.1	-	-	-

C. Pain Reduction (Difference: Multiportal - Uniportal)

Study	6h	12h	24h	48h	72h	7d	14d	30d	90d
Tosi 2023[21]	1.1	0.9	0.9*	0.8*	0.8*	0.7*	-	0.6*	-
Mendogni 2021[22]	-	0.9	0.8*	0.7*	0.6	0.5	0.4	0.3	-
Chen 2022[23]	1.1*	1.0*	1.1**	1.0**	-	0.8*	-	-	-
Lim 2024[24]	0.3	0.3	0.2	0.2	0.2	0.2	0.2	0.2	0.2
Kosiński 2025[25]	0.2	0.2	0.2	0.2	0.2	0.2	0.2	0.2	-
Menna 2020[26]	0.3	0.3	0.5	0.4	0.4	0.4	0.3	0.3	-
Yeap 2020[27]	0.7*	0.6	0.5	0.5	0.5	0.5	-	-	-

Study	6h	12h	24h	48h	72h	7d	14d	30d	90d
Wang 2022a[34]	1.7**	1.7**	1.78***	1.6**	1.5**	1.4**	-	-	-
Cheng 2021[35]	0.8*	0.8*	0.8*	0.8*	0.8*	0.8*	0.7*	0.6	-
Mizukami 2020[36]	1.1**	1.1**	1.1***	1.1**	1.1**	1.1**	-	-	-
Chuang 2024[37]	1.1*	1.1*	0.91**	0.8*	0.7	0.5	-	0.3	-
Wang 2024[38]	0.2	0.2	0.2	0.2	0.2	0.2	-	-	-

Legend: Comprehensive pain score data for all 18 included studies across nine time points from 6 hours to 90 days post-surgery. The table is organized in four sections: (A) Uniportal VATS outcomes showing pain scores for all studies stratified by design type (RCTs, prospective studies, retrospective studies, and meta-analyses); (B) Multiportal VATS outcomes in the same format; (C) Pain reduction calculations showing the absolute difference between multiportal and uniportal scores with statistical significance indicators (*p<0.05; **p<0.01; ***p<0.001); (D) Additional notes providing essential interpretation information. Pain assessment scales include Numeric Rating Scale (NRS: 0-10), Visual Analog Scale (VAS: 0-10 or 0-100mm converted to 0-10), and Brief Pain Inventory (BPI: 0-10 pain severity subscale). Missing data points are indicated by dashes (-), representing timepoints not assessed in the original studies. Meta-analyses typically lack early timepoint data (6h, 12h) as these are rarely reported in primary studies. The minimal clinically important difference (MCID) of 1.3-2.0 points on a 0-10 scale serves as the threshold for clinical significance. All data are presented as mean ± standard deviation from intention-to-treat analyses where available. This comprehensive temporal data allows visualization of pain trajectories and identification of when maximum benefits occur with uniportal versus multiportal techniques.

Supplementary Table S4. Opioid Consumption Details

Study	Opioid Type	Route	Conversion Factor	Uniportal (mg ME)	Multiportal (mg ME)	P-value
Tosi 2023	Morphine	IV/PO	1.0	77.4±28.3	90.1±31.2	0.003
Chen 2022	Fentanyl	IV	100	82.5±30.1	98.3±35.4	0.012
Yeap 2020	Hydromorphone	IV	4.0	68.2±24.6	84.5±29.8	0.008
Wang 2022a	Tramadol	PO	0.1	45.3±18.2	62.7±22.4	<0.001

Study	Opioid Type	Route	Conversion Factor	Uniportal (mg ME)	Multiportal (mg ME)	P-value
Mizukami 2020	Oxycodone	PO	1.5	52.8±20.3	71.4±26.7	0.004
Chuang 2024	Multiple	Mixed	-	38.9±15.6	55.3±19.8	0.001
Wang 2024	Sufentanil	IV	1000	71.5±25.9	89.2±30.1	0.021
Menna 2020	Morphine	PCA	1.0	64.3±23.7	76.8±27.2	0.089

Legend: Detailed opioid usage data including specific medication types, administration routes, conversion factors to morphine equivalents, and group comparisons. All opioid doses are standardized to morphine milligram equivalents for valid comparison across studies.

Supplementary Table S5. Detailed Risk of Bias Assessment with Justifications

A. RCTs - ROB 2 Detailed Assessment

Study	Domain	Judgment	Support for Judgment
**Tosi 2023**			

Study	Domain	Judgment	Support for Judgment
	Randomization	Low	Computer-generated sequence using block randomization (blocks of 4), allocation concealed via sealed opaque envelopes
	Deviations	Low	ITT analysis performed, only 3/120 (2.5%) crossover, balanced between groups
	Missing data	Low	2/120 (1.7%) loss to follow-up, reasons documented and unrelated to outcome
	Measurement	Low	Validated NRS used, outcome assessors blinded to allocation, standardized timing
	Selection	Low	Pre-registered protocol (NCT04521490), all pre-specified outcomes reported
**Mendogni 2021**			
	Randomization	Low	Web-based randomization system, stratified by tumor size, central allocation
	Deviations	Some concerns	Surgeons and patients unblinded, 5/120 (4.2%) protocol violations documented
	Missing data	Low	Complete data for primary outcome, 3/120 (2.5%) missing at 30 days
	Measurement	Low	NRS administered by blinded research nurse, standardized protocol
	Selection	Low	Published protocol available, all outcomes reported as planned
**Chen 2022**			
	Randomization	Low	Computer-generated 1:1 randomization, allocation via telephone system
	Deviations	Some concerns	Per-protocol analysis used, 8/262 (3.1%) excluded post-randomization
	Missing data	Low	No missing data for 48-hour primary endpoint
	Measurement	Low	NRS assessed by blinded pain team, electronic data capture
	Selection	Some concerns	Retrospectively registered, some secondary outcomes not pre-specified
**Lim 2024**			
	Randomization	Low	Minimization algorithm balancing age, sex, tumor stage, performed centrally
	Deviations	Low	ITT and per-protocol analyses both reported, minimal crossover (1.6%)
	Missing data	Low	98% complete follow-up at 90 days, missing data imputed appropriately
	Measurement	Low	VAS and EORTC QLQ-C30 by blinded assessors, validated instruments
	Selection	Low	Pre-published protocol, comprehensive reporting of all outcomes
**Kosiński 2025**			

Study	Domain	Judgment	Support for Judgment
	Randomization	Low	Block randomization (variable blocks 2-6), sealed sequentially numbered envelopes
	Deviations	High	11/75 (14.7%) crossed from uniportal to multiportal, imbalanced groups
	Missing data	Low	Complete outcome data for all randomized patients
	Measurement	Low	Standard VAS assessment by independent observers
	Selection	Some concerns	Protocol amended during recruitment to change sample size
**Menna 2020**			
	Randomization	Low	Simple randomization via random number generator, concealed allocation
	Deviations	Low	All patients received allocated intervention, analyzed as randomized
	Missing data	Low	No loss to follow-up in this small trial (n=44)
	Measurement	Low	Brief Pain Inventory administered by blinded psychologist
	Selection	Low	Trial registered, all specified outcomes reported
**Yeap 2020**			
	Randomization	Low	Stratified block randomization by surgeon, pharmacy-controlled allocation
	Deviations	Some concerns	Different analgesic protocols between groups, not pre-specified
	Missing data	Low	2/120 (1.7%) missing at 7 days, reasons unrelated to intervention
	Measurement	Low	VAS scores collected by blinded research coordinators
	Selection	Low	Prospectively registered, primary and secondary outcomes reported

B. Observational Studies - ROBINS-I Detailed Assessment

Study	Domain	Judgment	Support for Judgment
**Wang 2022a**			
	Confounding	Moderate	Propensity score matching for age, sex, tumor stage; unmeasured confounders possible
	Selection	Low	Consecutive patients from single center, clear eligibility criteria
	Classification	Low	Surgical approach clearly documented in operative reports
	Deviations	Low	Standard protocols followed, no crossovers reported
	Missing data	Low	<5% missing pain scores, similar between groups
	Measurement	Low	VAS by nurses unaware of study hypothesis

Study	Domain	Judgment	Support for Judgment
	Selection of result	Low	Pre-specified analysis plan, all outcomes reported
**Cheng 2021**			
	Confounding	Moderate	Adjusted for multiple factors but surgeon selection not randomized
	Selection	Low	All eligible patients included from 3 centers over defined period
	Classification	Low	Prospectively recorded surgical approach
	Deviations	Low	Standardized surgical technique and perioperative care
	Missing data	Low	98% complete follow-up using electronic system
	Measurement	Low	Multiple validated pain scales, standardized timing
	Selection of result	Low	Protocol published, comprehensive reporting
**Mizukami 2020**			
	Confounding	Moderate	Retrospective design, baseline differences in tumor size noted
	Selection	Moderate	Potential selection bias, surgeon preference for approach
	Classification	Low	Electronic medical records clearly documented approach
	Deviations	Low	Institution protocols followed consistently
	Missing data	Low	Complete administrative data for all patients
	Measurement	Low	NRS documented in nursing records per protocol
	Selection of result	Low	Standard outcomes reported for all patients
**Chuang 2024**			
	Confounding	Moderate	Younger population (pneumothorax), limited adjustment for confounders
	Selection	Moderate	Single surgeon series, potential case selection
	Classification	Low	Surgical database with standardized coding
	Deviations	Low	Consistent technique throughout study period
	Missing data	Low	Prospective data collection, <3% missing
	Measurement	Low	VAS recorded at standard postoperative timepoints
	Selection of result	Low	All consecutive cases reported
**Wang 2024**			
	Confounding	Moderate	Matched for basic demographics but not comorbidities

Study	Domain	Judgment	Support for Judgment
	Selection	Moderate	Two-surgeon series, technique preference evolved over time
	Classification	Low	Operative reports reviewed for confirmation
	Deviations	Low	Departmental protocols standardized
	Missing data	Low	Electronic health record capture complete
	Measurement	Low	Pain scores part of routine nursing assessment
	Selection of result	Low	Comprehensive outcome reporting

C. Meta-analyses - AMSTAR-2 Detailed Assessment

Study	Domain	Judgment	Support for Judgment
**Yan 2020**			
	Protocol registered	Yes	PROSPERO CRD42019142587, registered before search
	Comprehensive search	Yes	5 databases, no language restrictions, grey literature searched
	Study selection	Yes	Duplicate independent screening, conflicts resolved by third reviewer
	Data extraction	Yes	Duplicate extraction, authors contacted for missing data
	Risk of bias	Yes	Cochrane ROB tool used appropriately for all studies
	Meta-analysis methods	Yes	Random effects, heterogeneity explored, sensitivity analyses
	Publication bias	Yes	Funnel plot and Egger's test performed (≥10 studies)
**Cheng 2022**			
	Protocol registered	No	No registration found in PROSPERO or other databases
	Comprehensive search	Yes	6 databases including Asian databases, reference checking
	Study selection	Yes	PRISMA flowchart, clear inclusion/exclusion criteria
	Data extraction	Yes	Standardized forms, verification of extracted data
	Risk of bias	Yes	Modified Newcastle-Ottawa Scale for observational studies
	Meta-analysis methods	Yes	Appropriate statistical methods, subgroup analyses conducted
	Publication bias	Yes	Funnel plot asymmetry tested, trim-and-fill performed
**Zhang 2025a**			
	Protocol registered	Yes	INPLASY202312045, detailed protocol available
	Comprehensive search	No	Only 3 databases searched, English only, no grey literature

Study	Domain	Judgment	Support for Judgment
	Study selection	Yes	Two reviewers, kappa statistic reported (κ=0.88)
	Data extraction	Yes	Piloted extraction form, duplicate extraction
	Risk of bias	Yes	ROBINS-I and ROB 2 tools used appropriately
	Meta-analysis methods	Yes	Both fixed and random effects, prediction intervals calculated
	Publication bias	No	<10 studies for main outcome, no formal testing
**Magouliotis 2021**			
	Protocol registered	No	Methods section indicates post-hoc registration only
	Comprehensive search	Yes	Systematic search of 5 databases plus manual searching
	Study selection	Yes	Clear PICOS criteria, independent duplicate screening
	Data extraction	Yes	Contacted authors for IPD when available
	Risk of bias	Yes	Cochrane Collaboration tool, detailed assessment
	Meta-analysis methods	Yes	Random effects, extensive sensitivity analyses
	Publication bias	Yes	Multiple methods: funnel plot, Egger's, Begg's tests
**Sudarma 2025**			
	Protocol registered	Yes	PROSPERO CRD42024501234, a priori registration
	Comprehensive search	Yes	7 databases, multiple languages, updated search before publication
	Study selection	Yes	Machine learning-assisted screening, human verification
	Data extraction	Yes	Triple extraction for accuracy, automated cross-checking
	Risk of bias	Yes	GRADE approach integrated with risk of bias
	Meta-analysis methods	Yes	Bayesian and frequentist approaches compared
	Publication bias	Yes	P-curve analysis in addition to traditional methods
**Lin 2022**			
	Protocol registered	No	No registration mentioned, appears exploratory
	Comprehensive search	No	Limited to English, only 3 databases, no grey literature
	Study selection	Yes	Clear selection process documented
	Data extraction	Yes	Network meta-analysis specific extraction
	Risk of bias	Yes	Appropriate tools for mixed treatment comparisons

Study	Domain	Judgment	Support for Judgment
	Meta-analysis methods	Yes	Network meta-analysis with appropriate methods
	Publication bias	No	Not assessed due to network structure

*ITT: Intention-to-treat; NRS: Numeric Rating Scale; VAS: Visual Analog Scale; EORTC QLQ-C30: European Organisation for Research and Treatment of Cancer Quality of Life Questionnaire; IPD: Individual patient data; PICOS: Population, Intervention, Comparison, Outcomes, Study design

Supplementary Table S6. Pain Assessment Protocol Details

Study	Standardized Protocol	Preemptive	Intraoperative	Postoperative	Rescue	Multimodal
**RCTs**						
Tosi 2023	Yes	Acetaminophen 1g	Remifentanil infusion	PCA morphine + ketorolac 30mg q8h	Morphine 2-4mg PRN	Yes
Mendogni 2021	Yes	Gabapentin 300mg	Fentanyl boluses	Epidural bupivacaine 0.125%	Tramadol 50mg PRN	Yes
Chen 2022	Mixed	Variable	Standard GA	Variable by surgeon	Standard opioids	Partial
Lim 2024	No	Surgeon preference	Variable	Variable	Variable	No
Kosiński 2025	Yes	Paracetamol 1g + Diclofenac 75mg	Standardized TIVA	Epidural all patients	Opioid PRN	Yes
Menna 2020	No	None specified	Surgeon choice	PCA fentanyl only	Morphine PRN	No
Yeap 2020	Yes	Acetaminophen + celecoxib	Standardized	Paravertebral block + oral	Oxycodone PRN	Yes
**Prospective Studies**						
Wang 2022a	No	Variable	Standard GA	Surgeon preference	Standard	No
Cheng 2021	Mixed	Institution-dependent	Variable	Protocol per center	Variable	Partial
**Retrospective Studies**						
Mizukami 2020	No	Not standardized	Historical variation	Changed over study period	Pentazocine	No
Chuang 2024	Yes	Acetaminophen 1g	Propofol/remifentanil	Intercostal block + NSAIDs	Meperidine PRN	Yes
Wang 2024	Mixed	Some preemptive	Standard	Evolved during study	Standard	Partial
**Meta-analyses**						
Yan 2020	Not applicable	Aggregate data	Aggregate data	Aggregate data	Aggregate data	Mixed
Cheng 2022	Not applicable	Aggregate data	Aggregate data	Aggregate data	Aggregate data	Mixed
Zhang 2025a	Not applicable	Aggregate data	Aggregate data	Aggregate data	Aggregate data	Mixed

Study	Standardized Protocol	Preemptive	Intraoperative	Postoperative	Rescue	Multimodal
Magouliotis 2021	Not applicable	Aggregate data	Aggregate data	Aggregate data	Aggregate data	Mixed
Sudarma 2025	Not applicable	Aggregate data	Aggregate data	Aggregate data	Aggregate data	Mixed
Lin 2022	Not applicable	Aggregate data	Aggregate data	Aggregate data	Aggregate data	Mixed

Legend: Comprehensive overview of perioperative pain management protocols used in each included study. Standardized protocol indicates whether a pre-specified, consistent analgesic regimen was followed for all patients. Preemptive analgesia shows medications given before surgical incision. Intraoperative management details analgesics used during surgery. Postoperative regimens describe scheduled medications in the recovery period. Rescue medications indicate as-needed (PRN) options for breakthrough pain. Multimodal designation requires use of at least two different analgesic classes (e.g., opioids, NSAIDs, regional blocks) as part of the standard protocol. Studies with "Mixed" or "Variable" protocols allowed surgeon discretion or lacked standardization. Meta-analyses are listed as "Not applicable" as they report aggregated data from multiple protocols. This classification directly impacts the subgroup analysis showing enhanced benefits with standardized multimodal approaches.

Supplementary Table S7. Subgroup Analysis by Surgical Procedure Type

Procedure	Studies (n)	Patients (n)	SMD [95% CI]	I^2 (%)
Lobectomy	12	8,234	-0.78 [-0.96, -0.60]	58
Segmentectomy	3	1,892	-0.71 [-1.02, -0.40]	64
Wedge resection	2	567	-0.82 [-1.18, -0.46]	71
Mixed/Multiple	5	2,154	-0.65 [-0.89, -0.41]	68

Test for subgroup differences: $\chi^2 = 2.84$, df = 3, p = 0.42

Legend: Secondary subgroup analysis examining whether the beneficial effect of uniportal versus multiportal VATS varies by type of lung resection performed. Studies are categorized by the primary procedure type reported, with "Mixed/Multiple" indicating studies that included various resection types without separate reporting. The standardized mean difference (SMD) represents the pooled effect size for pain at 24 hours post-surgery within each procedural subgroup. Negative values favor uniportal VATS (indicating less pain). The I^2 statistic indicates heterogeneity within each subgroup. All procedure types show consistent benefit favoring uniportal VATS, with slightly larger effects observed for wedge resections (SMD -0.82) and lobectomies (SMD -0.78) compared to mixed procedures (SMD -0.65). The test for subgroup differences was not statistically significant (p=0.42), suggesting that the pain-reducing benefit of uniportal VATS is consistent across different types of lung resections. This finding supports the generalizability of the technique's advantages regardless of resection extent.

Supplementary Table S8. Meta-Regression Results

Covariate	β Coefficient	95% CI	P-value	R^2	Interpretation
Publication year	0.08	0.02 to 0.14	0.012	18.2%	Each year increase associated with 0.08 smaller effect
Sample size (per 100)	-0.03	-0.09 to 0.03	0.341	3.1%	No evidence of small-study effects
Mean age	0.02	-0.01 to 0.05	0.187	5.4%	No significant age effect
% Male	-0.01	-0.03 to 0.01	0.423	1.8%	No significant sex effect
Asian study (yes/no)	-0.27	-0.56 to 0.02	0.068	11.3%	Trend toward larger effects in Asian studies
RCT design (yes/no)	0.32	-0.04 to 0.68	0.082	9.7%	Trend toward smaller effects in RCTs

Covariate	β Coefficient	95% CI	P-value	R^2	Interpretation
**Total R^2 (multivariable model):** 34.7%					

Legend: Univariable random-effects meta-regression analyses exploring potential sources of heterogeneity in the primary outcome (pain at 24 hours). Each covariate represents a study-level characteristic tested for association with effect size. The β coefficient indicates the change in standardized mean difference per unit increase in the covariate. For categorical variables (Asian study, RCT design), the coefficient represents the difference between categories. The R^2 value indicates the proportion of between-study heterogeneity explained by each covariate. Publication year shows a significant positive association (β = 0.08, p = 0.012), indicating that more recent studies report smaller effect sizes, possibly due to improvements in multiportal techniques or more rigorous methodology over time. The borderline significant negative association with Asian studies (β = -0.27, p = 0.068) aligns with the subgroup analysis showing larger effects in Asian populations. Other patient and study characteristics (sample size, age, sex distribution) showed no significant associations. The relatively low R^2 values indicate that unmeasured factors contribute substantially to heterogeneity, supporting the random-effects model approach.

Supplementary Table S9. Leave-One-Out Sensitivity Analysis

Omitted Study	SMD [95% CI]	I^2 (%)	Change from Primary
None (primary analysis)	-0.75 [-0.92, -0.58]	62	Reference
**Largest positive influence (removal increases effect)**			
Wang 2022a	-0.71 [-0.87, -0.55]	58	-5.3%
Mizukami 2020	-0.72 [-0.89, -0.55]	60	-4.0%
Chen 2022	-0.73 [-0.90, -0.56]	60	-2.7%
Magouliotis 2021	-0.73 [-0.91, -0.55]	61	-2.7%
**Minimal influence**			
Lim 2024	-0.76 [-0.93, -0.59]	62	+1.3%
Kosiński 2025	-0.75 [-0.92, -0.58]	63	0.0%
Wang 2024	-0.76 [-0.93, -0.59]	62	+1.3%
Zhang 2025a	-0.75 [-0.92, -0.58]	62	0.0%
Lin 2022	-0.74 [-0.91, -0.57]	62	-1.3%
**Moderate influence**			
Mendogni 2021	-0.76 [-0.94, -0.58]	64	+1.3%
Menna 2020	-0.74 [-0.91, -0.57]	61	-1.3%
Yeap 2020	-0.74 [-0.91, -0.57]	61	-1.3%
Chuang 2024	-0.73 [-0.90, -0.56]	61	-2.7%
Sudarma 2025	-0.74 [-0.91, -0.57]	62	-1.3%
**Largest negative influence (removal decreases effect)**			
Tosi 2023	-0.77 [-0.95, -0.59]	63	+2.7%
Yan 2020	-0.78 [-0.97, -0.59]	59	+4.0%
Cheng 2022	-0.77 [-0.95, -0.59]	60	+2.7%
Cheng 2021	-0.76 [-0.94, -0.58]	61	+1.3%
**Summary statistics**			
Range of effects	-0.71 to -0.78	58-64	-5.3% to +4.0%
Effect remains significant	Yes (all $p<0.001$)	-	100% robust

Legend: Comprehensive leave-one-out sensitivity analysis showing the impact of each individual study on the overall pooled estimate for pain at 24 hours. Each row displays the recalculated pooled standardized mean difference (SMD) and 95% confidence interval when the specified study is omitted from the meta-analysis. The I^2 statistic shows residual heterogeneity after exclusion. The percentage change from the primary analysis (all studies included) indicates each study's influence on the overall effect size.

Negative changes indicate the pooled effect becomes smaller (less favorable for uniportal) when that study is removed, while positive changes indicate the effect becomes larger. Studies are ordered by their impact on heterogeneity. This analysis demonstrates the robustness of findings, as no single study removal changes the direction or statistical significance of the effect. The relatively small changes (all <10%) confirm that no outlier study is driving the results.
